# Prediction of microRNAs Associated with Human Diseases Based on Weighted *k* Most Similar Neighbors

**DOI:** 10.1371/journal.pone.0070204

**Published:** 2013-08-08

**Authors:** Ping Xuan, Ke Han, Maozu Guo, Yahong Guo, Jinbao Li, Jian Ding, Yong Liu, Qiguo Dai, Jin Li, Zhixia Teng, Yufei Huang

**Affiliations:** 1 Key Laboratory of Database and Parallel Computing of Heilongjiang Province, School of Computer Science and Technology, Heilongjiang University, Harbin, China; 2 School of Computer and Information Engineering, Harbin University of Commerce, Harbin, China; 3 Department of Computer Science and Engineering, Harbin Institute of Technology, Harbin, China; 4 School of Information Science and Technology, Heilongjiang University, Harbin, China; 5 Department of Electrical and Computer Engineering, University of Texas, San Antonio, Texas, United States of America; CSIR-Institute of Microbial Technology, India

## Abstract

**Background:**

The identification of human disease-related microRNAs (disease miRNAs) is important for further investigating their involvement in the pathogenesis of diseases. More experimentally validated miRNA-disease associations have been accumulated recently. On the basis of these associations, it is essential to predict disease miRNAs for various human diseases. It is useful in providing reliable disease miRNA candidates for subsequent experimental studies.

**Methodology/Principal Findings:**

It is known that miRNAs with similar functions are often associated with similar diseases and vice versa. Therefore, the functional similarity of two miRNAs has been successfully estimated by measuring the semantic similarity of their associated diseases. To effectively predict disease miRNAs, we calculated the functional similarity by incorporating the information content of disease terms and phenotype similarity between diseases. Furthermore, the members of miRNA family or cluster are assigned higher weight since they are more probably associated with similar diseases. A new prediction method, HDMP, based on weighted *k* most similar neighbors is presented for predicting disease miRNAs. Experiments validated that HDMP achieved significantly higher prediction performance than existing methods. In addition, the case studies examining prostatic neoplasms, breast neoplasms, and lung neoplasms, showed that HDMP can uncover potential disease miRNA candidates.

**Conclusions:**

The superior performance of HDMP can be attributed to the accurate measurement of miRNA functional similarity, the weight assignment based on miRNA family or cluster, and the effective prediction based on weighted *k* most similar neighbors. The online prediction and analysis tool is freely available at http://nclab.hit.edu.cn/hdmpred.

## Introduction

MicroRNAs (miRNAs) are a set of short (21∼24 nt) non-coding RNAs that play important roles in gene regulation by targeting mRNAs for cleavage or translational repression [1], [2]. MiRNAs are involved in many important biological processes including cell differentiation, proliferation, and apoptosis [3]. Furthermore, accumulating evidence indicates miRNAs are associated with various human diseases [4]–[7].

Identifying the relationship between miRNAs and diseases by experimental methods, such as microarray profiling and qRT-PCR, has been proven successful. However, the false positive microarray results can be caused by the different melting temperatures of miRNAs [8]–[11]. Furthermore, the experimental cost is greatly increased by the probe design [12]–[14]. Therefore, development of computational methods that predict the reliable disease-related miRNA candidates is a valuable complement to experimental studies [15]–[22]. So far, little work is available in predicting disease miRNAs.

First, it was shown that functionally related miRNAs tend to be associated with phenotypically similar diseases [18]. Jiang *et al.* constructed the miRNA network by establishing a functional relationship of two miRNAs based on their target genes. Their target genes are predicted by the target prediction programs PITA [23] and TargetScan [24]. They integrated the miRNA network with a phenome network to infer potential miRNA-disease associations. In addition, Jiang *et al.* further improved the calculation of concordance score between a miRNA and a given disease [19]. However, the high false positive in miRNA target predictions [25] restrains the efficacy of Jiang's methods.

Second, it was reported that if miRNAs are associated with a similar regulatory pattern in the same type of disease, their target genes may share common functional characteristics [20]. Based on these results, Li *et al.* prioritized the miRNAs for a specific disease by estimating the functional consistency score (FCS) among their target genes and the known target genes associated with the disease [21]. The target genes of these miRNAs are predicted by the target prediction programs miRanda [26], PicTar [27] and TargetScan. FCS method was applied to 11 human diseases including breast cancer, lung cancer and etc. However, besides the high false positive in miRNA target predictions, the limited known disease-related target genes also restrain the method's usage for the 11 diseases. For other important human diseases, such as heart failure, the method is unable to provide their prediction results.

Third, it is observed that miRNAs with similar functions are often associated with similar diseases and vice versa [8], [20], [28], [29]. The functional similarity of two miRNAs is successfully estimated by the semantic similarity of their associated two groups of diseases [20]. On the basis of the calculated similarities, RWRMDA constructed a miRNA similarity network. The new miRNA-disease associations are predicted based on random walking on the network [22]. However, the association information between the miRNAs passed by a walker and a specific disease is overlooked. Furthermore, RWRMDA does not consider the characteristics of the members from miRNA family or cluster.

In this study, we improved the functional similarity estimation method developed in [20] by further considering the information content of disease terms and phenotype similarity between diseases. Subsequently, the members of miRNA family or cluster are assigned higher weight since they are more probably associated with similar diseases. At last, we presented an effective prediction algorithm based on weighted *k* most similar neighbors (HDMP). HDMP's prediction performance is evaluated by performing 5-fold cross validation and another validation based on an updated dataset. The results indicate HDMP achieves better performance than the existing methods.

## Materials and Methods

### Disease miRNAs prediction based on weighted *k* most similar neighbors

For a specific disease *d*, we refer to the experimentally validated miRNAs associated with *d* as the *labeled miRNAs*. The others which have no evidence to validate that they are associated with *d* until now are referred to as the *unlabeled miRNAs*. As the unlabeled miRNAs are probably associated with *d*, our goal is to rank the unlabeled miRNAs according to their possibilities of associating with *d*. To achieve this goal, we correlate an unlabeled miRNA *u* with a relevance score *Score*(*u*). A greater *Score*(*u*) means higher possibility that *u* is associated with *d*. We then rank all the unlabeled miRNAs according to their relevance scores and select the top ranked miRNAs as potential *d*-related candidates.

**Figure 1:**
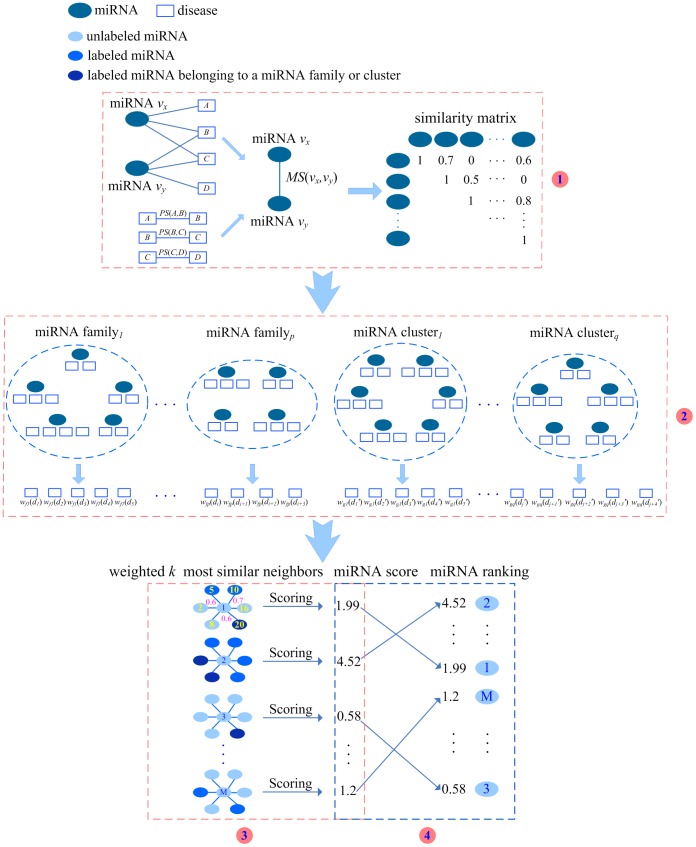
Process of predicting disease *d*-related candidates. Step 1: calculate the functional similarity of any two miRNAs and construct a symmetric functional similarity matrix. Step 2: assign the members of miRNA family or cluster higher weight. Step 3: calculate the relevance score of each unlabeled miRNA. Step 4: rank all the unlabeled miRNAs according to their scores and select the top ranked miRNAs as potential candidates.

The process of predicting *d*-related candidates includes four steps, as shown in Figure 1. First, the functional similarity of any two miRNAs is calculated by incorporating the semantic similarity and the phenotype similarity between diseases, and then a symmetric functional similarity matrix is constructed. Second, the members of each miRNA family or cluster are assigned higher weight according to the miRNA-disease association information in the family or cluster. Third, the relevance score of each unlabeled miRNA is estimated by considering the functional similarities of its weighted *k* most similar neighbors and the distribution information of the labeled miRNAs in these neighbors. Fourth, all the unlabeled miRNAs are ranked by their relevance scores. The miRNAs with higher ranks are potential *d*-related candidates. Our proposed prediction method is referred to as HDMP.

### MiRNA functional similarity measurement

The prediction performance of HDMP is highly dependent on accurate miRNA functional similarity measurement. It is observed that miRNAs with similar functions are often associated with similar diseases and vice versa [8], [20], [28], [29]. Therefore, Wang *et al.* proposed to estimate functional similarity of two miRNAs by measuring the semantic similarity of their associated diseases [20]. In this section we give a brief overview of Wang's measurement. In the next section, we pointed out its inadequacy and further proposed the improved estimation strategy.

**Figure 2:**
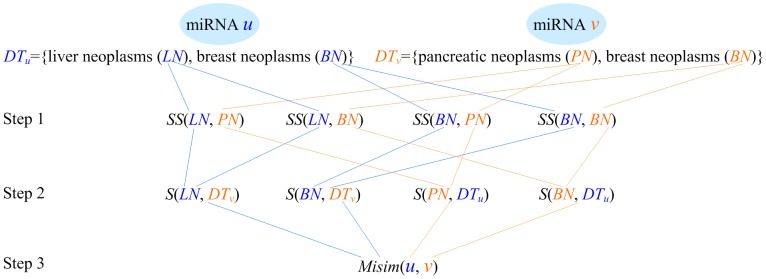
Measuring the functional similarity between miRNA *u* and *v*.

Assume that *DT_u_* and *DT_v_* represent a group of diseases associated with the miRNA *u* and *v*, respectively, and, for example, *DT_u_* = {*liver neoplasms* (*LN*), *breast neoplasms* (*BN*)} and *DT_v_* = {*pancreatic neoplasms* (*PN*), *breast neoplasms* (*BN*)}. The similarity between *DT_u_* and *DT_v_* is calculated as the functional similarity between *u* and *v*, denoted as *Misim*(*u*, *v*). As shown in Figure 2, Wang's measurement process contains three steps.

**Figure 3:**
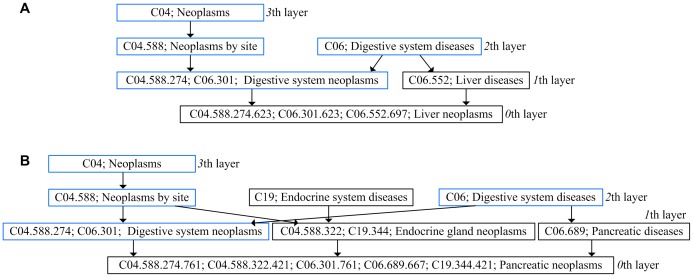
The disease DAGs of liver neoplasms and pancreatic neoplasms. (a) DAG of liver neoplasms. (b) DAG of pancreatic neoplasms. The nodes in blue are the disease terms shared by the two DAGs.

First, the semantic similarity of any two diseases *d_u_* and *d_v_* (*d_u_*∈*DT_u_*, *d_v_*∈*DT_v_*) is calculated, such as *SS*(*LN*, *PN*). Two diseases *LN* (*liver neoplasms*) and *PN* (*pancreatic neoplasms*) are represented by directed acyclic graph (DAG), as shown in Figure 3. In the DAG of *LN*, ‘*liver neoplasms*’ in the *0*th layer is the most specific disease term and therefore its contribution to its own semantic value is defined as 1. Since ‘*Digestive system neoplasms*’ in the *1*th layer is a more general denomination, its contribution is multiplied by the semantic contribution factor (Δ = 0.5). Wang *et al.* defined the factor Δ to differentiate the semantic contribution values of disease terms in different layers. ‘*Neoplasms by site*’ in the *2*th layer is even more general than ‘*Digestive system neoplasms*’ and its contribution is further factored as 0.5×0.5. Thus, the semantic value of *LN* is *DV*(*LN*) = 1.0 (1.0 is the semantic contribution value of ‘*liver neoplasms*’)+0.5 (‘*digestive system neoplasms*’)+0.5×0.5 (‘*neoplasms by site*’)+0.5×0.5×0.5 (‘*neoplasms*’)+0.5 (‘*liver diseases*’)+0.5×0.5 (‘*digestive system diseases*’) = 2.625. In the same way, the semantic value of *PN* is *DV*(*PN*) = 3.375. Suppose *T_LN_* is the set of all ancestor nodes of ‘*liver neoplasms*’ including node ‘*liver neoplasms*’ itself and *t* represents a blue node shared by the two diseases and *t*∈*T_LN_*∩*T_PN_*. The sum of semantic contributions of all the blue nodes in Figure 3A is ∑*D*(*LN*, *t*) = 0.5 (‘*digestive system neoplasms*’)+0.5×0.5 (‘*neoplasms by site*’)+0.5×0.5×0.5 (‘*neoplasms*’)+0.5×0.5 (‘*digestive system diseases*’) = 1.125. Similarly, ∑*D*(*PN*, *t*) in Figure 3B is 1.125. The semantic similarity of these two diseases, *SS*(*LN*, *PN*), is calculated as
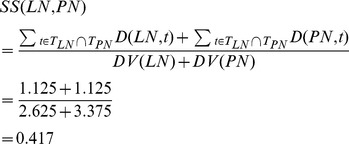
(1)Also, we have *SS*(*LN*, *BN*) = 0.357, *SS*(*BN*, *PN*) = 0.3125, and *SS*(*BN*, *BN*) = 1.

Second, the similarity between one of diseases associated with miRNA *u*, such as *LN*, and the group of diseases associated with miRNA *v*, *DT_v_* = {*PN*, *BN*}, is denoted as *S*(*LN*, *DT_v_*) and calculated as
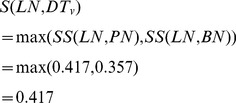
(2)Therefore, *S*(*BN*, *DT_v_*) = 1, *S*(*PN*, *DT_u_*) = 0.417, and *S*(*BN*, *DT_u_*) = 1.

Third, the similarity between two groups of diseases, *DT_u_* and *DT_v_*, is calculated as the similarity of their associated two miRNAs, i.e., the functional similarity of *u* and *v*. It is denoted as *Misim*(*u*, *v*) and defined as follows.
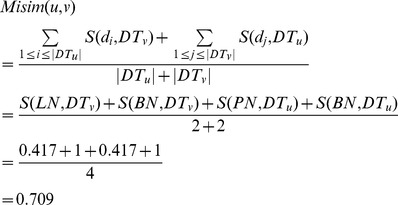
(3)


### Incorporating information content of disease terms and disease phenotype similarity

Wang's measurement has been proved successful in estimating the functional similarity of two miRNAs. However, we found its inadequacy. As shown in Figure 3, the farther a disease term is from the *0*th layer, the more general the disease term is and the less semantic contribution it has. Wang *et al.* defined the semantic contribution of a disease term in the *k*th layer as 0.5*^k^*. Thus, the disease terms in the same layer (e.g., ‘*digestive system neoplasms*’ and ‘*liver diseases*’ in the *1*th layer in Figure 3A) have the same semantic contribution value (0.5).

However, we found that ‘*digestive system neoplasms*’ appears in 40 disease DAGs, such as DAG of *esophageal neoplasms* and DAG of *liver neoplasms*. ‘*liver diseases*’ appears in 73 disease DAGs, such as DAG of *liver failure* and DAG of *liver cirrhosis*. Obviously, the former is more specific than the latter since the former appears in less DAGs. The semantic contribution of the former should be higher than the latter. Therefore, it is less accurate to assign the same contribution value to the disease terms of the same layer in Wang's measurement.

Intuitively, the more specific a disease term is, the more informative it is for calculating the functional similarity. Therefore, we calculate the information content of per disease term as its semantic contribution. In this way, a more specific disease term has a greater semantic contribution value. Given that the likelihood of a disease term *t* appearing in all the disease DAGs is denoted as *p*(*t*), the information content of *t*, *IC*(*t*), can be quantified as the negative log of the likelihood, and *IC*(*t*) = −*log*[*p*(*t*)]. The information content of all the 4577 disease terms was calculated and available at our web site. Thus, the semantic value of ‘*liver neoplasms*’, *DV*(*LN*), is updated as 10.160 (10.160 is the value of *IC*(*liver neoplasms*))+6.838 (*IC*(*digestive system neoplasms*))+4.453 (*IC*(*neoplasms by site*))+2.785 (*IC*(*neoplasms*))+6.116 (*IC*(*liver diseases*))+3.961 (*IC*(*digestive system diseases*)) = 34.313. In the same way, *DV*(*PN*) is 46.566. The semantic similarity *SS*(*LN*, *PN*) is calculated as
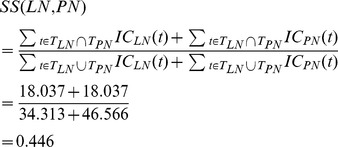
(4)Therefore, *SS*(*LN*, *BN*) = 0.215, *SS*(*BN*, *PN*) = 0.182, and *SS*(*BN*, *BN*) = 1.

Obviously, a greater semantic similarity revealed that two diseases are more likely similar with each other. In addition, the similarity of two diseases is closely related to their phenotypes. We obtained the similarity between any two of 5080 disease phenotypes from the literature [30]. The phenotype similarity between two diseases, such as *A* and *B*, is denoted as *PS*(*A*, *B*). In order to incorporate the semantic similarity and the phenotype similarity, the similarity between *A* and *B* is defined as *DS*(*A*, *B*).
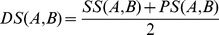
(5)Thus, *DS*(*LN*, *PN*) is calculated as follows.
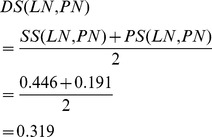
(6)Therefore, *DS*(*LN*, *BN*) = 0.295, *DS*(*BN*, *PN*) = 0.287, and *DS*(*BN*, *BN*) = 1.

Next, the similarity between *LN* and *DT_v_*, *S*(*LN*, *DT_v_*), is updated as
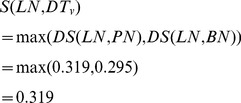
(7)Also, we have *S*(*BN*, *DT_v_*) = 1, *S*(*PN*, *DT_u_*) = 0.319, and *S*(*BN*, *DT_u_*) = 1. Thus, the similarity between miRNA *u* and *v* is denoted as *MS*(*u*,*v*) and calculated as follows.
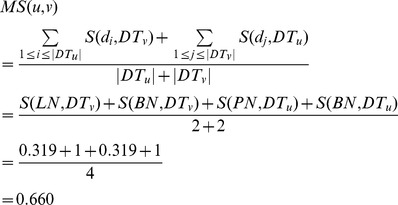
(8)


### Assignment of weight based on miRNA families or clusters

It was reported that the members of miRNA family or cluster are more likely to associate with the similar diseases [8], [20], [31]. Therefore, these miRNAs are assigned higher weight and the assignment strategy is described as follows.

#### Assignment of weight based on miRNA families

The homologous miRNAs are gathered into the same miRNA family by RFam [32]. The seed regions (normally 2–8*th* nucleotide from the 5′ end of miRNA) of miRNA sequences of the same family are almost identical. Since the seed of a miRNA is commonly required to be perfectly complementary to the target mRNAs for cleavage or translational repression, the members of the same family likely regulate a common set of mRNA targets. Hence, it is more likely that they are associated with the similar diseases [20], [31]. Assume *u* is an unlabeled miRNA and *v* is one of its *k* most similar neighbors. Also, assume *v* is associated with disease *d*. Furthermore, *u* and *v* belong to a same family. As far as *v* is concerned, *u* is more possibly associated with *d*. At this time, *v* is assigned higher weight. In the future, the weight will be multiplied by the functional similarity between *u* and *v* as the subscore of *v*, detailed in section ‘Calculation of the relevance scores of miRNA candidates’.

We download the information of miRNA families from the latest miRNA database miRBase 19. The 474 miRNAs involved in the 4379 miRNA-disease associations cover 52 families. For the *i*th (1≤*i*≤52) family, the rate of *d*-related miRNAs accounting for its size is defined as
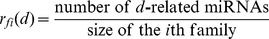
(9)For instance, assume there are 10 miRNAs in the *i*th miRNA family. 6 of 10 miRNAs are associated with *d*. Thus, we have *r_fi_*(*d*) = 6/10 = 0.6. The greater *r_fi_*(*d*) means that most of miRNAs in the family have been associated with *d*. The remaining miRNAs are more likely to associate with *d*.

Assume two miRNAs do not belong to the same family. The weight of these two miRNAs with respect to *d* is viewed as 1. Assume another two miRNAs belong to the *i*th family and some miRNAs of the family are associated with *d*. The weight of the miRNAs in the *i*th family with respect to *d* should be greater than 1 and it is defined as
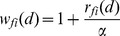
(10)where *α* is a factor for adjusting the weight. To find a suitable *α* value, the different *α* values from 1 to 10 are tested by performing 5-fold cross validation. Figure S1A shows HDMP achieved better prediction performance when *α* = 4 than other values. Therefore, we set *α* as 4 in this study.

**Figure 4:**
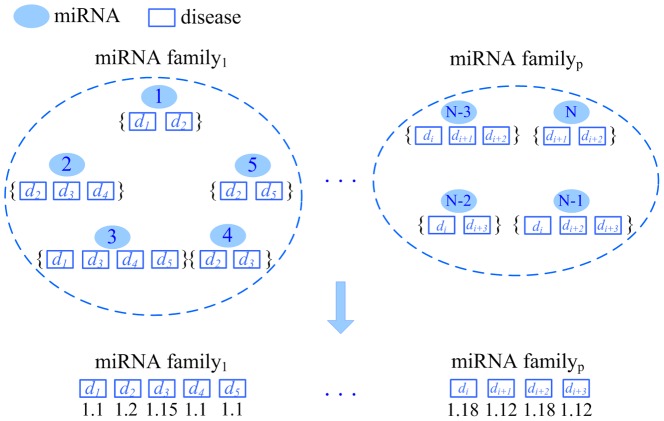
Assigning weight for the members of a miRNA family according to their associations with a group of diseases.

For each family, we calculate the weight of its members for each disease involved in the family. The calculation process is illustrated by miRNA family 1. As shown in Figure 4, assume there are *p* families and family 1 is composed of 5 miRNAs, including miRNA 1, 2, 3, 4, and 5. Assigning weight for family 1 includes the following 3 steps.

1. All the diseases that are associated with the members of family 1 are collected to form the disease set *S_1_* = {*d*_1_,*d*_2_,*d*_3_,*d*_4_,*d*_5_}.
2. We collect the miRNAs associated with disease *d_i_* (1≤*i*≤5), respectively. For instance, *d_1_* is associated with miRNA 1 and 3. *d_2_* is associated with miRNA 1, 2, 4, and 5. *d_3_* is associated with miRNA 2, 3, and 4. *d_4_* is associated with miRNA 2 and 3. *d_5_* is associated with miRNA 3 and 5.
3. The weight of the miRNAs in family 1 with respect to disease *d_i_* (1≤*i*≤5) is calculated. For instance, *d_2_* is associated with miRNA 1, 2, 4, and 5. Family 1 is composed of 5 miRNAs. Thus, *d_2_*-related miRNAs account for four fifth of family 1 and *r_f1_*(*d_2_*) = 4/5. The weight about *d_2_* is *w_f1_*(*d_2_*) = 1+*r_f1_*(*d_2_*)/*α* = 1+(4/5)/*α* = 1+(4/5)/4 = 1.2.

Repeating above 3 steps, the weight can be calculated for family 2, …, and family *p*, respectively.

#### Assignment of weight based on miRNA clusters

It has been reported that miRNAs are often found in genomic clusters [33]. The clustered miRNAs are usually transcribed together and more likely associated with the similar diseases [8], [20]. Therefore, if the unlabeled miRNA *u* and one of its neighbor *v* belong to a same cluster, *v* is assigned higher weight.

We download the chromosomal coordinates of human miRNAs from miRBase 19. Wang *et al.* confirm that the clustered miRNAs located within 20 kb of genomic location are more likely to associate with the similar diseases [20]. Therefore, we merge the miRNAs whose distances are within 20 kb into a same cluster. The 474 miRNAs involved in the 4379 miRNA-disease associations cover 58 clusters. For the *i*th (1≤*i*≤58) cluster, the rate of disease *d*-related miRNAs accounting for its size is defined as
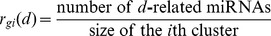
(11)The greater *r_gi_*(*d*) means that most of miRNAs in the *i*th cluster have been associated with *d*. The remaining miRNAs are more possibly associated with *d*. The weight of two miRNAs not belonging to the same cluster is viewed as 1. Assume there are another two miRNAs belonging to the *i*th cluster and some miRNAs of the cluster are associated with *d*. The weight of the miRNAs in the *i*th cluster with respect to *d* should be greater than 1 and it is defined as
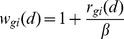
(12)where *β* is a factor for adjusting the weight. The different *β* values from 1 to 10 were investigated by the experiments. HDMP achieved the highest prediction performance when *β* is 4 (Figure S1B).

### Calculation of the relevance scores of miRNA candidates

For a specific disease *d*, to reliably estimate the relevance score of an unlabeled miRNA *u*, its *k* most similar neighboring miRNAs are observed. Assume miRNA *v* is one of the *k* neighbors and it is associated with *d*. Since *u* and its neighbor *v* have higher functional similarity, they are more possibly associated with a group of similar diseases. Thus, as far as *v* is concerned, *u* is also possibly associated with *d*. The greater the functional similarity between *u* and *v*, the higher the possibility that *u* is associated with *d*. Therefore, the functional similarity is considered when estimating the relevance score of *u*.

We correlate each of *k* neighbors with a subscore. The subscores of *k* neighbors are accumulated as the relevance score of *u*. The 3 combinations of *u* and *v* are listed as following.

1. MiRNA *u* and its neighbor *v* are not in the same miRNA family or cluster. Also, *v* is associated with *d*. For instance, as shown in the *3*th part of Figure 1, miRNA 1 is an unlabeled miRNA. Since its neighbor 5 is associated with *d*, 1 is possibly associated with *d*. The greater functional similarity between 1 and 5, *MS*(1, 5), means that 1 is more likely to associate with *d*. Thus, with respect to 1, the subscore of 5 is assigned to *MS*(1, 5) = 0.6.
2. MiRNA *u* and its neighbor *v* belong to the same miRNA family or cluster. Also, *v* is associated with *d*. In Figure 1, since the neighbor 20 is associated with *d*, 1 is possibly associated with *d*. Furthermore, as both 1 and 20 are in the *i*th family, they are more likely to associate with *d*. Therefore, to assign the subscore of 20, we consider the functional similarity between 1 and 20. At the same time, the weight of these two miRNAs in this family is considered. The subscore of 20 is *MS*(1, 20)×*w_fi_*(*d*) = 0.6×1.15 = 0.69. In addition, if two miRNAs not only belong to a family but also belong to a cluster, both the weight based on this family and that based on this cluster are multiplied by their functional similarity as the subscore.
3. The neighbor *v* has no evidence to validate that it is associated with *d*. For instance, miRNA 2 is a neighbor of 1 and 2 is not associated with *d*. As far as 2 is concerned, it is very little possibility that 1 is associated with *d*. At this time, the subscore of 2 is assigned to 0.

The sum of subscores of *k* neighbors is calculated as the relevance score of *u*. As shown in Figure 1, 3 miRNAs (5, 10, and 20) are associated with disease *d* in the 6 most similar neighbors of 1. The functional similarities between 1 and 5, 10, 20 are 0.6, 0.7, and 0.6 respectively. Furthermore, 1 and 20 are in the *i*th family and the weight *w_fi_*(*d*) is 1.15. At this time, the subscores of 5 and 10 are 0.6 and 0.7 respectively. The subscore of 20 is 0.6*1.15 = 0.69. Since 2, 8, 16 are not associated with *d*, all of their subscores are 0. Thus, the relevance score of 1 is denoted as *Score*(1) = 0.6+0.7+0.69 = 1.99. In this way, we calculate the relevance scores for all the unlabeled miRNAs.

**Figure 5:**
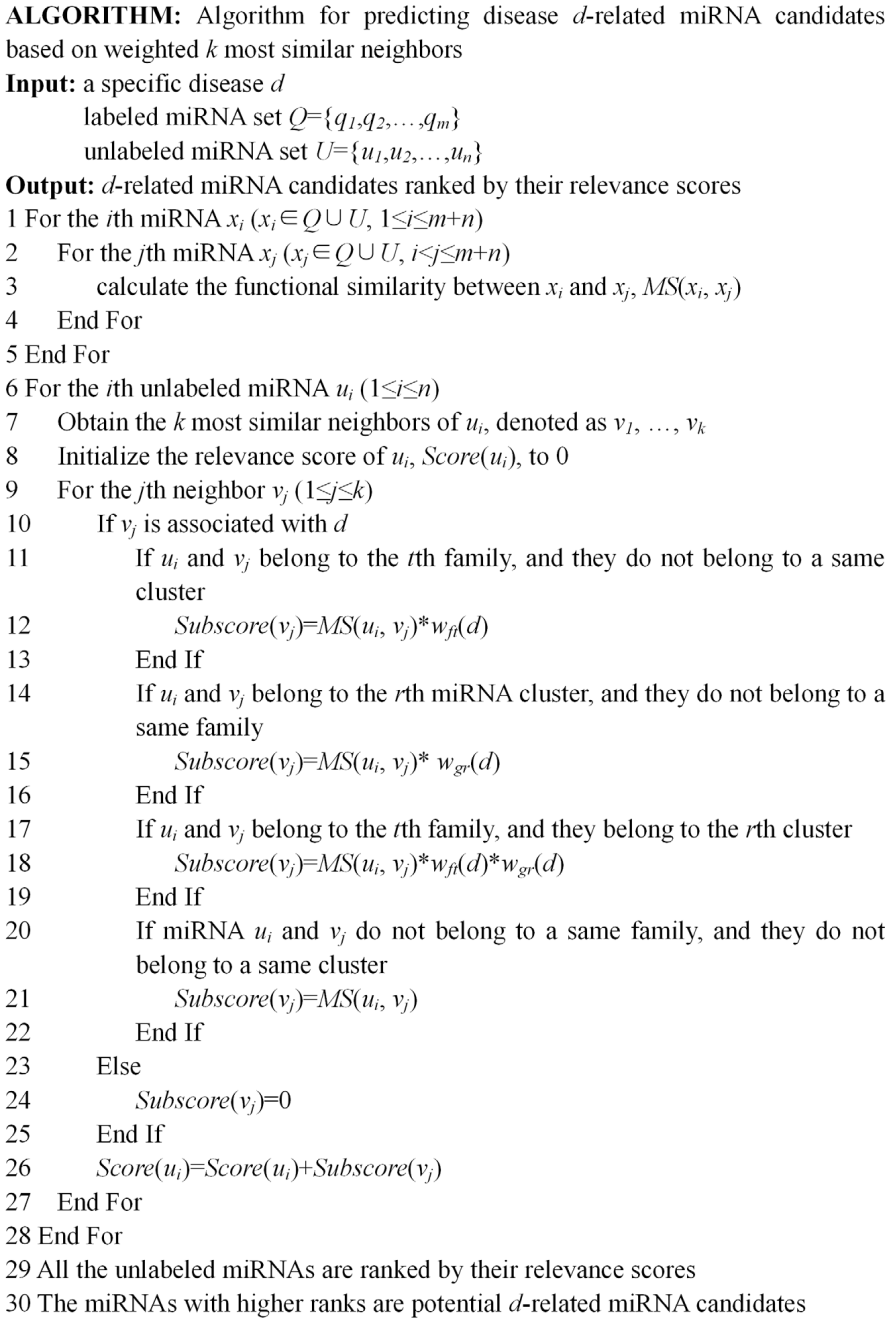
Algorithm of predicting the miRNA candidates associated with disease *d*.

For a specific disease *d*, assume the labeled miRNA set is *Q* = {*q_1_*,*q_2_*,…,*q_m_*}. The unlabeled miRNA set is *U* = {*u_1_*,*u_2_*,…,*u_n_*}. To determine the association possibility of an unlabeled miRNA *u* (*u*∈*U*) with *d*, the sum of subscores of weighted *k* neighbors most similar to *u* is calculated as its relevance score. The higher score means a more possible association between *u* and *d*. The algorithm of predicting *d*-related miRNA candidates is described in Figure 5.

The relevance score accumulation between an unlabeled miRNA and its neighbors is dependent on the parameter *k*. If *k* is too great, the noise data will be included, which will not contribute to improving the prediction performance. If *k* is too small, there is no sufficient data to accurately estimate the relevance scores. The different *k* values from 1 to 50 were investigated by the experiments. Figure S1C shows HDMP achieved the highest prediction performance when *k* is 20.

## Results and Discussion

### Data preparation

The human miRNA-disease associations were downloaded from the human miRNA-disease database HMDD [29]. Two versions (November-2010 Version and September-2012 Version) of HMDD associations were used in the experiments. Invalid miRNA-disease associations with incorrect disease names or miRNA names are filtered out. The correct disease names were downloaded from the National Library of Medicine (http://www.nlm.nih.gov/). The correct miRNA names were obtained from the latest miRNA database miRBase 19 [34]. After filtering, November-2010 Version contains 2076 associations between 338 miRNAs and 199 diseases, and September-2012 Version contains 4379 miRNA-disease associations between 474 miRNAs and 268 diseases. The similarity between any two of 5080 OMIM disease phenotypes was obtained from the literature [30]. Since the disease names of OMIM are named differently from those of MeSH, their mapping information was downloaded from the comparative toxicogenomics database [35].

### Prediction performance evaluation

To evaluate HDMP's ability of predicting disease miRNAs, *5*-*fold cross validation* was performed firstly. For a specific disease *d*, the labeled miRNAs of September-2012 Version are randomly divided into 5 subsets, 4 of which are used as known information to predict candidates, while the left out subset is used for testing. The *d*-related miRNA candidate pool consisted of all the unlabeled miRNAs and the labeled miRNAs used for testing. The relevance score of each unlabeled miRNA and that of each labeled miRNA in the pool are calculated. All these miRNAs are ranked by their relevance scores. The higher the labeled miRNAs are ranked, the better the prediction performance is.

If a labeled miRNA has higher rank than a given threshold, HDMP is considered to successfully predict it. By varying the threshold, the true positive rate (sensitivity) and the false positive rate (1-specificity) were calculated to obtain the receiver operating characteristic (ROC) curves. Sensitivity is the proportion of the labeled miRNAs successfully predicted accounting for all the labeled miRNAs in the pool. Specificity is the proportion of the unlabeled miRNAs which have lower ranks than the threshold accounting for all the unlabeled miRNAs. The area under the ROC curve (AUC) was calculated to demonstrate the prediction performance of HDMP. To obtain reliable evaluation result, we tested the 18 human diseases which are associated with at least 60 miRNAs respectively. As shown in Table 1, HDMP achieved the highest AUC with *pancreatic neoplasms*, and the lowest AUC with *lupus vulgaris*. The average AUC value for the 18 diseases is 0.825.

**Table 1:** Prediction results of HDMP and other methods for 5-fold cross validation.

	No. of associated miRNAs	AUC			
Disease name		HDMP	FCS method	Jiang's method	RWRMDA
Acute myeloid leukemia	60	0.822	0.575	0.526	0.635
Adenoviridae infections	68	0.686			0.605
Breast neoplasms	196	0.819	0.671	0.598	0.695
Colorectal neoplasms	128	0.785	0.612	0.603	0.688
Glioblastoma	86	0.887			0.638
Heart failure	118	0.797			0.642
Hepatocellular carcinoma	206	0.785		0.515	0.626
Lung neoplasms	119	0.899	0.718	0.667	0.697
Lupus vulgaris	60	**0.681**			0.603
Medulloblastoma	60	0.799		0.516	0.638
Melanoma	132	0.842	0.698	0.634	0.708
Ovarian neoplasms	107	0.836		0.531	0.673
Pancreatic neoplasms	95	**0.922**	0.664	0.609	0.712
Prostatic neoplasms	96	0.884	0.656	0.596	0.754
Renal cell carcinoma	88	0.828			0.649
Squamous cell carcinoma	67	0.812			0.682
Stomach neoplasms	77	0.866		0.577	0.691
Urinary bladder neoplasms	66	0.895	0.715	0.635	0.759

There are 8, 12, and 18 common diseases between HDMP and FCS method, Jiang's method, and RWRMDA, respectively. ‘No. of associated miRNAs’ indicates the number of miRNAs associated with a specific disease in September-2012 Version of HMDD.

In addition, the associations of November-2010 Version were used to construct HDMP. HDMP was applied to predict the set of associations added into HMDD between November-2010 and September-2012. The set of associations formed the updated dataset. The validation based on the dataset is called *updated dataset validation*. There are 9 diseases each of which is associated with at least 60 miRNAs in November-2010. The 9 diseases were tested to further evaluate the prediction performance of HDMP. As shown in Table 2, the highest AUC was obtained with *pancreatic neoplasms*, and the lowest AUC was obtained with *hepatocellular carcinoma*. The average AUC for the 9 diseases is 0.726.

**Table 2:** Prediction results of HDMP and other methods for updated dataset validation.

	No. of associated miRNAs	No. of new added miRNAs	AUC			
Disease name			HDMP	FCS method	Jiang's method	RWRMDA
Breast neoplasms	101	66	0.671	0.602	0.538	0.628
Hepatocellular carcinoma	65	119	**0.649**		0.609	0.612
Colonic neoplasms	64	13	0.651			0.601
Heart failure	101	16	0.751			0.631
Lung neoplasms	92	27	0.754	0.612	0.565	0.655
Melanoma	105	21	0.791	0.648	0.535	0.609
Ovarian neoplasms	74	30	0.727		0.519	0.611
Pancreatic neoplasms	60	38	**0.797**	0.609	0.554	0.735
Stomach neoplasms	65	19	0.741		0.576	0.622

There are 4, 7, and 9 common diseases between HDMP and FCS method, Jiang's method, and RWRMDA, respectively. ‘No. of associated miRNAs’ indicates the number of miRNAs associated with a specific disease in November-2010 Version of HMDD. ‘No. of new added miRNAs’ indicates the number of miRNAs associated with a specific disease which are added into HMDD between November-2010 and September-2012.

### Importance for improving miRNA similarity measurement and incorporating miRNA family or cluster

To validate the importance for improving miRNA functional similarity measurement, two HDMP's instances were constructed based on our measurement and Wang's measurement respectively. The 5-fold cross validation was performed to evaluate the performance of these two instances. The former achieved higher AUC values for all the 18 human diseases (Table S1). The minimum AUC increase is 1.6% for *adenoviridae infections* and the maximum one is 3.7% for *medulloblastoma*. For the 18 diseases, the AUC is increased by 2.2% on average. It demonstrates our measurement is effective for improving the prediction performance. In addition, the prediction instance based on Wang's measurement achieves decent performance. It further confirms the prediction method based on weighted *k* neighbors is sufficient to ensure the prediction accuracy.

In addition, we constructed three prediction instances and listed their prediction results in Table S2. The first instance was constructed only based on *k* most similar neighbors without considering miRNA family and cluster. The others further incorporated miRNA family and cluster respectively. For the 18 diseases, the average AUC of the second instance is 2.9% greater than the first one. The third instance is also increased by 2.8% on average. It shows the importance of incorporating miRNA family and cluster during construction of the efficient prediction instance.

### Comparison with FCS method, Jiang's method, and RWRMDA

We first compared HDMP with FCS method proposed in [21]. FCS method ranked the miRNA candidates based on the functional consistency between miRNA target genes and disease-related genes. While FCS method ranked disease miRNA candidates for 11 diseases, our HDMP method ranked for 18 diseases. The 5-fold cross validation was performed on their 8 common diseases. In addition, there are 4 common diseases for the updated dataset validation. The ranked miRNAs by FCS method were downloaded from the web site (http://bioinfo.hrbmu.edu.cn/CMP). The results over 5-fold cross validation (Table 1) and those over updated dataset validation (Table 2) show that HDMP is more accurate than FCS method. The average AUC value for 8 common diseases is increased by 19.5% and that for 4 common diseases is increased by 13.5%. We measured the statistical significance of the difference in their AUCs by paired *t*-test. The *p*-values are reported in Table 3. Clearly, HDMP performs significantly better than FCS method at the significance level 0.05.

**Table 3:** *p*-values obtained by paired *t*-testing the AUCs of HDMP and those of another prediction method.

Validating over different dataset	FCS method	Jiang's method	RWRMDA
HDMP over 5-fold cross validation	2.337e-06	1.155e-10	2.592e-11
HDMP over updated dataset validation	0.006	0.0004	0.0002

As mentioned before, FCS method is dependent on the predicted miRNA target genes. However, it is difficult to obtain highly accurate target genes although FCS method integrated the results of 3 target prediction programs to minimize the false positive. HDMP is based on the accurate measurement of miRNA functional similarity and effective prediction process by observing weighted *k* most similar neighbors. Thus, HDMP achieves better prediction performance.

Second, we compared HDMP with Jiang's method presented in [18], where the potential miRNA-disease associations were inferred based on the human phenome-miRNAnome network. Jiang's another method presented in [19] can not be compared since its source code and web service are unavailable. The disease description in Jiang's method comes from the Online Mendelian Inheritance in Man (OMIM) database [36]. Due to the slight differences between the disease names of OMIM and those of MeSH, there are no exact correspondences for 6 of the 18 diseases for 5-fold cross validation. Also, there are no corresponding disease names for 2 of the 9 diseases for updated dataset validation. Consequently, we compared the results of HDMP and those of Jiang's method for their common diseases (Table 1 and Table 2). The *p*-values by paired *t*-test are listed in Table 3. It is clear that the prediction performance of HDMP is significantly better than that of Jiang's method. The average AUC value for 12 common diseases is increased by 24.9% and that for 7 common diseases is increased by 17.6%. Jiang *et al.* constructed the miRNAnome network based on the predicted miRNA targets. These targets were obtained by simply merging the results of 2 target prediction programs. Thus, the high false positive in the merged targets has a great effect on the performance of Jiang's method.

Third, RWRMDA was originally constructed by using the 1395 miRNA-disease associations in the earlier version of HMDD (September, 2009). Unfortunately, the source code of RWRMDA provided by its web site is not available currently. To compare with RWRMDA, we implement RWRMDA based on 5-fold cross validation and updated dataset validation, respectively. The restart probability *r* of RWRMDA is set to 0.9 suggested by the experiments in [22]. The *p*-values by paired *t*-test are listed in Table 3. It indicates that HDMP performed significantly better than RWRMDA. The average AUC value for 18 diseases over 5-fold cross validation is increased by 15.3% and that for 9 diseases over updated dataset validation is increased by 9.2%. As mentioned before, RWRMDA predicted the disease miRNAs by random walking on the miRNA similarity network. However, when a walker moving from a miRNA to one of its neighbors, RWRMDA overlooked whether the miRNA is associated with *d* or not. It is not good for more specifically predicting *d*-related miRNAs. HDMP considers the *k* most similar neighbors and the distribution information of the known *d*-related miRNAs in these neighbors. Furthermore, HDMP incorporates the weight information of miRNA family or cluster. Therefore, HDMP achieved better performance.

**Figure 6:**
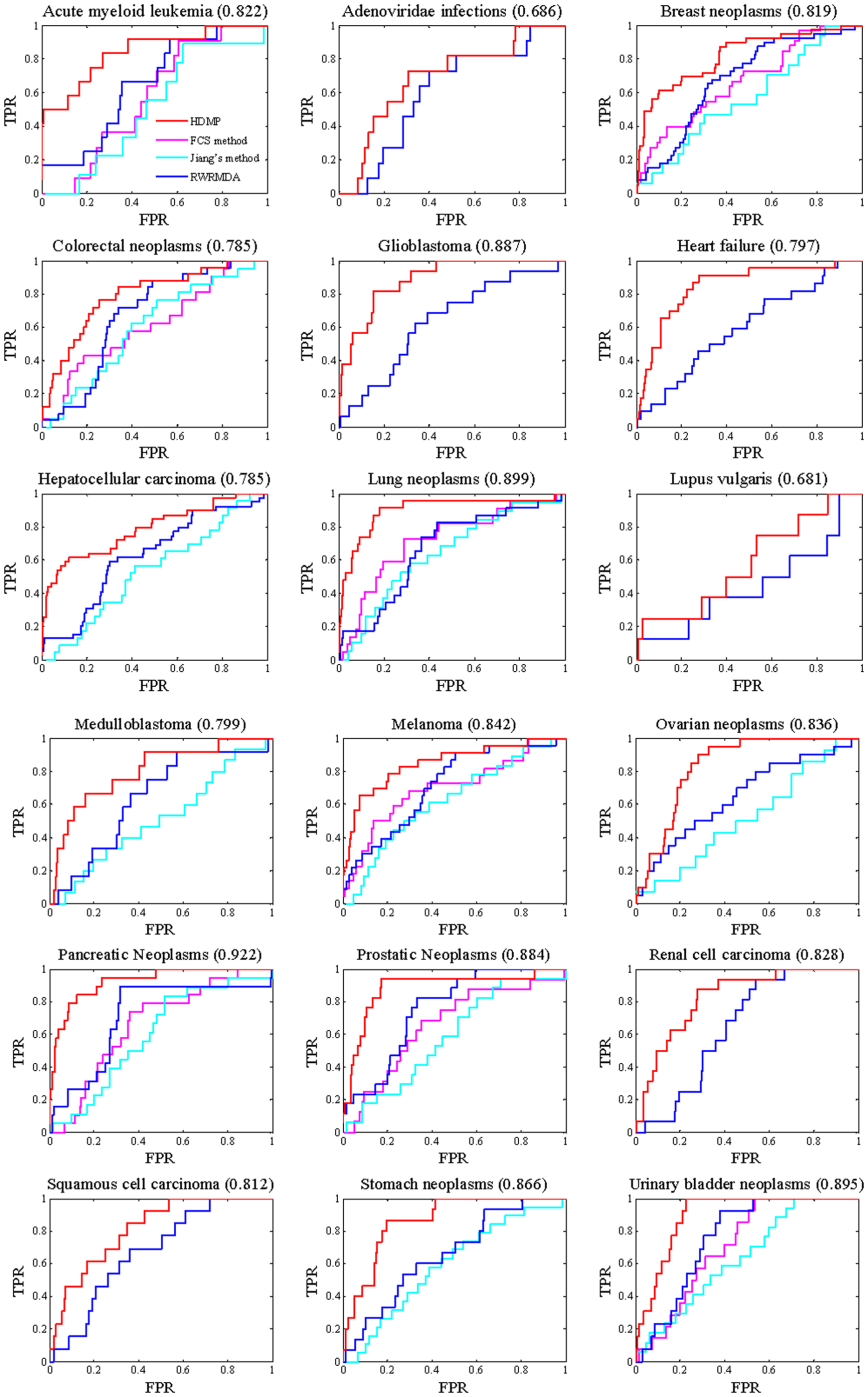
ROC curves of HDMP and other methods for 5-fold cross validation. Each value in bracket is the area under HDMP's ROC curve.

**Figure 7:**
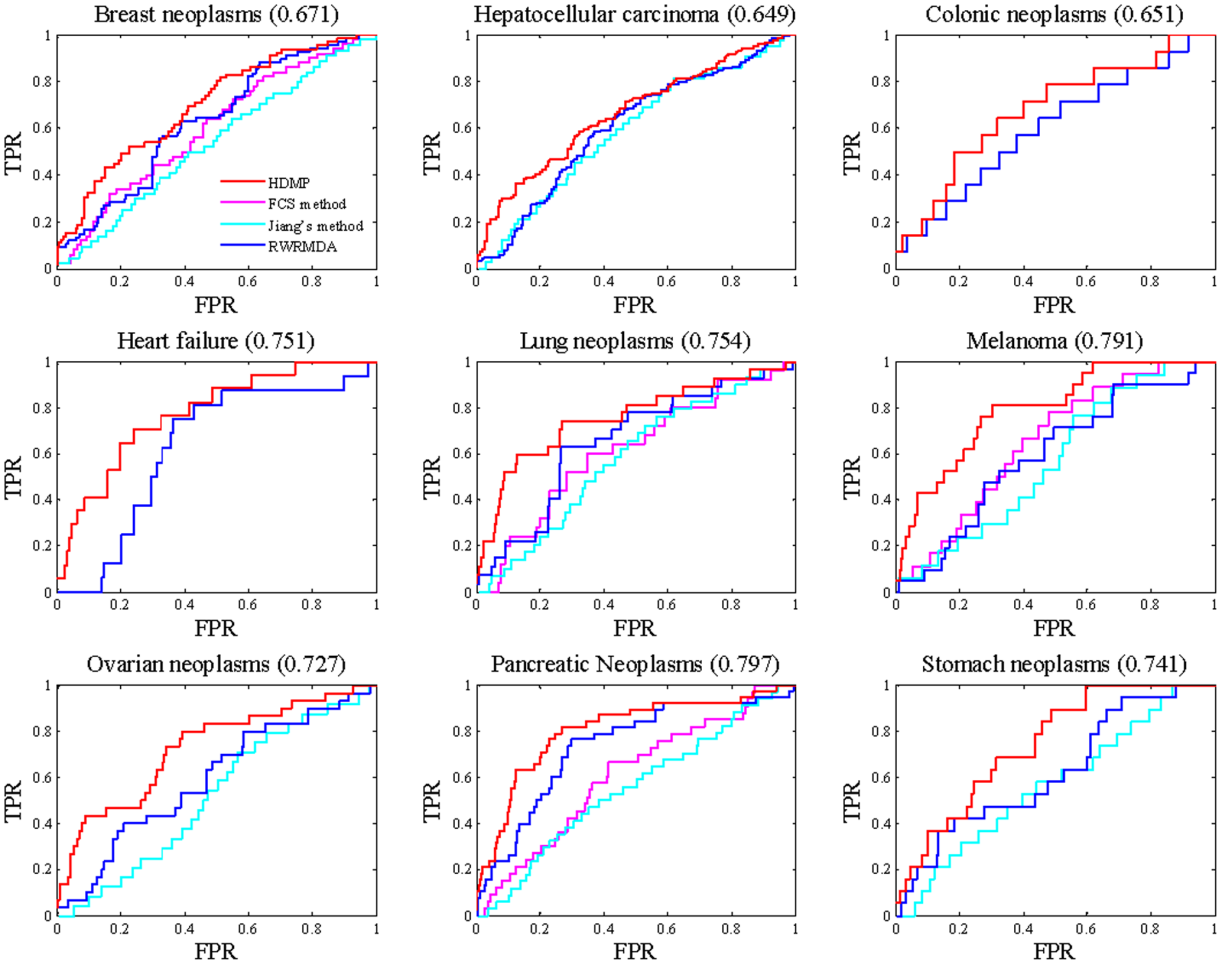
ROC curves of HDMP and other methods for updated dataset validation. Each value in bracket is the area under HDMP's ROC curve.

The ROC curves for 5-fold cross validation and those for updated dataset validation are demonstrated in Figure 6 and 7 respectively. RWRMDA performed better than FCS method and Jiang's method for most of diseases. HDMP outperformed all the previous methods. It indicates that HDMP can successfully recover the known disease miRNAs. In addition, for all the prediction methods, their overall performance over 5-fold cross validation is better than that over updated dataset validation. The primary reason is that the number of labeled miRNAs in the training dataset for the former is greater than that for the latter.

### Case studies: prostatic neoplasms, breast neoplasms, and lung neoplasms

To further demonstrate the ability of HDMP to uncover potential disease-related miRNA candidates, we present the case studies of prostatic neoplasms, breast neoplasms, and lung neoplasms. Many researchers have shown that miRNAs play critical role in the three diseases. Due to space limitations, we only provide a comprehensive analysis of the prostatic neoplasms-related candidates.

HDMP predict the candidates by using the miRNA-disease associations in the earlier version of HMDD (1 January 2012). The newly reported prostatic neoplasms-related miRNAs after January 1 2012 are used to validate the predicted candidates. Furthermore, the miRNA-disease relevant databases “miR2Disease” [37] and “dbDEMC” [38] are also used to confirm the candidates.

The top 50 candidates in the ranked list are illustrated in Table 4, and detailed in Table S3. First, during the period from January 2012 to September 2012, HMDD has been updated three times. There are 24 newly reported prostatic neoplasms-related miRNAs. 9 of 50 miRNAs are supported by the newly reported miRNAs. It indicates that HDMP can discover potentially important prostatic neoplasms-related miRNAs.

**Table 4:** The top 50 prostatic neoplasms-related miRNA candidates.

Rank	MiRNA name	Description	Rank	MiRNA name	Description
1	hsa-mir-429	higher RWRMDA (No. 2), higher Jiang (No. 1)	26	hsa-mir-24	dbDEMC, miR2Disease
2	hsa-mir-9	dbDEMC, literature	27	hsa-mir-29c	dbDEMC
3	hsa-mir-142	higher FCS (No. 48)	28	hsa-mir-30b	dbDEMC, miR2Disease
4	hsa-let-7i	dbDEMC	29	hsa-mir-125a	dbDEMC, miR2Disease
5	hsa-mir-155	dbDEMC	30	hsa-mir-18b	higher RWRMDA (No. 45)
6	hsa-mir-34b	dbDEMC	31	hsa-mir-20b	Higher FCS (No. 5)
7	hsa-mir-19a	dbDEMC	32	hsa-mir-30d	dbDEMC
8	hsa-mir-92a	HMDD, miR2Disease	33	hsa-mir-451	literature
9	hsa-mir-210	miR2Disease	34	hsa-mir-152	dbDEMC
10	hsa-mir-19b	dbDEMC, miR2Disease	35	hsa-mir-215	dbDEMC
11	hsa-mir-224	dbDEMC, miR2Disease	36	hsa-mir-130a	dbDEMC, HMDD
12	hsa-let-7f	dbDEMC, miR2Disease	37	hsa-mir-499	higher RWRMDA (No. 42)
13	hsa-mir-199b	dbDEMC, HMDD, miR2Disease	38	hsa-mir-206	dbDEMC
14	hsa-mir-181a	dbDEMC, miR2Disease	39	hsa-mir-192	dbDEMC
15	hsa-mir-29a	dbDEMC, HMDD, miR2Disease	40	hsa-mir-335	literature
16	hsa-let-7e	dbDEMC	41	hsa-mir-365	literature
17	hsa-mir-107	HMDD	42	hsa-mir-30a	miR2Disease
18	hsa-mir-18a	higher RWRMDA (No. 15), higher FCS (No. 92)	43	hsa-mir-302a	dbDEMC
19	hsa-let-7g	dbDEMC, miR2Disease	44	hsa-mir-212	literature
20	hsa-let-7b	dbDEMC, HMDD, miR2Disease	45	hsa-mir-372	dbDEMC
21	hsa-mir-150	dbDEMC, literature	46	hsa-mir-197	dbDEMC
22	hsa-mir-338	dbDEMC	47	hsa-mir-124	literature
23	hsa-mir-103	dbDEMC, miR2Disease	48	hsa-mir-378	HMDD
24	hsa-mir-15b	dbDEMC, HMDD	49	hsa-mir-26b	dbDEMC, miR2Disease
25	hsa-mir-31	dbDEMC, HMDD, miR2Disease	50	hsa-mir-542	higher RWRMDA (No. 25)

(1) ‘literature’ means that there is a literature to support that the miRNA is upregulated or downregulated in human prostatic neoplasm, as compared with normal prostatic tissue. (2) With analysis of the microarray data sets, a miRNA is considered to potentially have different express levels in prostatic cancer when compared to normal tissues. This kind of miRNAs is labeled by ‘dbDEMC’. (3) ‘HMDD’ means that a miRNA is a newly reported prostatic neoplasms-related miRNA which is collected by the latest version of HMDD. (4) ‘miR2Disease’ means that a miRNA is included in the manually curated miRNA-disease association database, miR2Disease. (5) ‘higher RWRMDA’ means a miRNA has higher rank in the ranked list of RWRMDA. (6) ‘higher FCS’ means a miRNA has greater functional consistency score (FCS) among their target genes and the known target genes associated with prostatic neoplasms. (7) ‘higher Jiang’ means a miRNA has higher rank in the ranked list of Jiang's method.

Second, miR2Disease is a manually curated database which provides a comprehensive resource of miRNA deregulation in various human diseases [37]. The current version of miR2Disease contains 3273 curated associations between 349 human miRNAs and 163 diseases. 17 of 50 miRNAs are included in miR2Disease. It indicates these miRNAs are deregulated in prostatic neoplasms, which confirms that they are really associated with prostatic neoplasms.

Third, several literatures confirm the 6 of 7 miRNAs are significantly upregulated or downregulated in human prostatic neoplasms versus normal prostatic tissue [39]–[43]. The remaining 1 miRNA is found to be up-regulated or down-regulated in the metastatic prostate cancer xenografts, relative to their non-metastatic counterparts [44]. HDMP successfully found these miRNAs due to their higher ranks.

Fourth, the database of differentially expressed miRNAs in human cancers, dbDEMC [38], is constructed to provide potential cancer-related miRNAs by *in silco* computing. The current version of dbDEMC contains 607 miRNAs which potentially have differential expression in 14 types of cancer, including prostatic cancer (malignant prostatic neoplasms). 33 of 50 miRNAs are contained in dbDEMC. These miRNAs are identified to be potentially upregulated or downregulated in prostatic cancer by using the significance analysis of the microarrays. It shows that the 33 miRNAs are more likely to participate in the prostatic cancer-related biological process.

Last but not least, 7 miRNAs have higher ranks in the ranked list of FCS method, Jiang's method and RWRMDA. Hsa-mir-429 is ranked No. 1 and No. 2 by Jiang's method and RWRMDA respectively. Hsa-mir-142, hsa-mir-18a, and hsa-mir-20b have greater functional consistency score (FCS) among their target genes and the known target genes associated with prostate neoplasms. Hsa-mir-18a, hsa-mir-18b, hsa-mir-499, and hsa-mir-542 are ranked No. 15, 45, 42, 25 by RWRMDA respectively. It indirectly confirms that the 7 miRNAs are more probably associated with prostatic neoplasms. All above analysis indicates the 50 miRNAs in Table 4 are potential prostatic neoplasms-related candidates.

In addition, the top 50 breast neoplasms-related candidates are demonstrated in Table S4. 19 of 50 miRNAs are confirmed to be associated with breast neoplasms by the newly reported miRNAs in HMDD. 8 miRNAs are validated by the database miR2disease. 3 miRNAs are supported to have deregulation in breast cancer by literatures [45]–[47]. The dbDEMC identified 39 miRNAs as potential miRNAs upregulated or downregulated in breast cancer (malignant breast neoplasms). The genes-to-systems breast cancer database, G2SBC [48], is usually used for assistant studying the breast cancer. For 2 miRNAs, at least 16 of top 100 their predicted target genes are breast cancer-related genes. It indicates that the 2 miRNAs are more probably associated with breast cancer. In addition, 2 miRNAs have higher ranks in the ranked list of FCS method and that of RWRMDA, which indirectly confirms they are potential breast neoplasms-related candidates.

The top 50 lung neoplasms-related candidates are listed in table S5. 6 of 50 miRNAs are confirmed to be associated with lung neoplasms by HMDD. 12 miRNAs are validated by the database miR2disease. 8 miRNAs are supported to be upregulated or downregulated in lung cancer by literatures [49]–[53]. The dbDEMC identified 31 miRNAs as potential deregulated miRNAs in lung cancer. 2 miRNAs are ranked higher by FCS method and RWRMDA. We have not found the evidence for only 2 miRNAs to confirm they are potentially associated with lung neoplasms. All above results demonstrate that HDMP is powerful in predicting potential disease-related miRNA candidates.

## Conclusions

A new prediction method based on weighted *k* most similar neighbors, HDMP, was developed for predicting disease miRNAs. We demonstrated the importance of accurately measuring miRNA functional similarity, incorporating weight information based on miRNA family or cluster, and considering the distribution information of a specific disease in achieving effective prediction result. A measurement strategy incorporating the information content of disease terms and phenotype similarity between diseases was proposed to accurately estimate the functional similarity of two miRNAs. The members of miRNA family or cluster are assigned higher weight according to their associations with a group of diseases. The functional similarity information and the distribution information of the disease *d*-related miRNAs in the *k* neighbors are incorporated to explore the possibility that a miRNA is associated with *d*.

HDMP has been compared with the existing prediction methods, including FCS method, Jiang's method, and RWRMDA. Both the results of 5-fold cross validation and those of the updated dataset validation demonstrated that HDMP has significantly higher accuracy in recovering the known disease miRNAs. The case studies of prostatic neoplasms, breast neoplasms, and lung neoplasms, further proved the ability of HDMP to uncover potential disease-related candidates. HDMP can provide reliable disease-related miRNA candidates for experimental research, which facilitates future studies of miRNA involvement in the pathogenesis of diseases.

## Supporting Information

Figure S1 **Prediction performance affected by α value, β value, and k value.**
(DOC)

Table S1 **Prediction results of HDMP with different functional similarity measurements.**
(DOC)

Table S2 **Prediction results for incorporating miRNA family and cluster respectively.**
(DOC)

Table S3 **The top 50 prostatic neoplasms-related miRNA candidates in the ranked list.** (1) ‘literature’ means that there is a literature to support that the miRNA is upregulated or downregulated in human prostatic neoplasm, as compared with normal prostatic tissue. (2) With analysis of the microarray data sets, a miRNA is considered to potentially have different express levels in prostatic cancer when compared to normal tissues. This kind of miRNAs is labeled by ‘dbDEMC’. (3) ‘HMDD’ means that a miRNA is a newly reported prostatic neoplasms-related miRNA which is collected by the latest version of human miRNA-disease database HMDD. (4) ‘miR2Disease’ means that a miRNA is included in the manually curated miRNA-disease association database, miR2Disease. (5) ‘higher RWRMDA’ means a miRNA has higher rank in the ranked list of RWRMDA. (6) ‘higher FCS’ means a miRNA has greater functional consistency score (FCS) among their target genes and the known target genes associated with prostatic neoplasms. (7) ‘higher Jiang’ means a miRNA has higher rank in the ranked list of Jiang's method.
(DOC)

Table S4 **The top 50 breast neoplasms-related miRNA candidates in the ranked list.** (1) ‘literature’ means that there is a literature to support that the miRNA is upregulated or downregulated in human breast neoplasm, as compared with normal breast tissue. (2) With analysis of the microarray data sets, a miRNA is considered to potentially have different express levels in breast cancer when compared to normal tissues. This kind of miRNAs is labeled by ‘dbDEMC’. (3) ‘HMDD’ means that a miRNA is a newly reported breast neoplasms-related miRNA which is collected by the latest version of human miRNA-disease database HMDD. (4) ‘miR2Disease’ means that a miRNA is included in the manually curated miRNA-disease association database, miR2Disease. (5) G2SBC is a genes-to-systems breast cancer database, which is usually used for assistant studying the breast cancer. ‘G2SBC’ means some of the top predicted target mRNAs of a miRNA are breast cancer-related genes. (6) ‘higher RWRMDA’ means a miRNA has higher rank in the ranked list of RWRMDA. (7) ‘higher FCS’ means a miRNA has greater functional consistency score (FCS) among their target genes and the known target genes associated with breast neoplasms.
(DOC)

Table S5 **The top 50 lung neoplasms-related miRNA candidates in the ranked list.** (1) ‘literature’ means that there is a literature to support that the miRNA is upregulated or downregulated in human lung neoplasm, as compared with normal lung tissue. (2) With analysis of the microarray data sets, a miRNA is considered to potentially have different express levels in lung cancer when compared to normal tissues. This kind of miRNAs is labeled by ‘dbDEMC’. (3) ‘HMDD’ means that a miRNA is a newly reported lung neoplasms-related miRNA which is collected by the latest version of human miRNA-disease database HMDD. (4) ‘miR2Disease’ means that a miRNA is included in the manually curated miRNA-disease association database, miR2Disease. (5) ‘higher RWRMDA’ means a miRNA has higher rank in the ranked list of RWRMDA. (6) ‘higher FCS’ means a miRNA has greater functional consistency score (FCS) among their target genes and the known target genes associated with lung neoplasms. (7) ‘higher Jiang’ means a miRNA has higher rank in the ranked list of Jiang's method. (8) ‘unconfirmed’ means there is no evidence to confirm that a miRNA is potentially associated with lung neoplasms.
(DOC)
