# Correction: Prediction of microRNAs Associated with Human Diseases Based on Weighted *k* Most Similar Neighbors

**DOI:** 10.1371/annotation/28592478-72f5-4937-919b-b2342d6ceda0

**Published:** 2013-08-23

**Authors:** Ping Xuan, Ke Han, Maozu Guo, Yahong Guo, Jinbao Li, Jian Ding, Yong Liu, Qiguo Dai, Jin Li, Zhixia Teng, Yufei Huang

Multiple funding organizations and grants were incorrectly omitted from the Funding Statement. The Funding Statement should read:

"The work was supported by the Natural Science Foundation of China (61070193, 60932008, 61172098, and 61271346), the Heilongjiang Province Foundation for Distinguished Young Scientists (JC201104), the Science and Technology Innovation Team Construction Project of Heilongjiang Province College (2013TD012), the Natural Science Foundation of Heilongjiang Province (F201119), the Science and Research Foundation of Heilongjiang Education Department (12521392, 12531154, 12531499, 12531476), the Young Innovative Talents Research Foundation of Harbin Science and Technology Bureau (2012RFQXG082, 2012RFQXS094, 2012RFQXG096), the Starting Research Foundation for Doctors of Harbin University of Commerce (13DL005), and the Open Project of Key Laboratory of Database and Parallel Computing in Heilongjiang Province (KLDP-0F-2012-02). Yufei Huang was supported by the National Science Foundation (CCF-0546345), the National Institute of Health (R01 CA096512), and the Qatar National Research Fund (09-874-3-235). The funders had no role in study design, data collection and analysis, decision to publish, or preparation of the manuscript." 

