# Correction: Prediction of microRNAs Associated with Human Diseases Based on Weighted *k* Most Similar Neighbors

**DOI:** 10.1371/annotation/a076115e-dd8c-4da7-989d-c1174a8cd31e

**Published:** 2013-09-23

**Authors:** Ping Xuan, Ke Han, Maozu Guo, Yahong Guo, Jinbao Li, Jian Ding, Yong Liu, Qiguo Dai, Jin Li, Zhixia Teng, Yufei Huang

In the E-mail list, guoyahong7225@163.com (YG) should be in front of maozuguo@hit.edu.cn (MG). The E-mail list should read:

"*E-mail: guoyahong7225@163.com (YG); maozuguo@hit.edu.cn (MG); yufei.huang@utsa.edu (YH)"

Posted by the authors of this article.

