# Supplementary material for: Prediction of microRNAs Associated with Human Diseases Based on Weighted k Most Similar Neighbors
Source: PLoS One. 2013 Aug 8;8(8):e70204. doi: 10.1371/journal.pone.0070204 (PMC3738541; doi:10.1371/journal.pone.0070204)
Supplement: Figure S1 — Prediction performance affected by α value, β value, and k value. (DOC) [file pone.0070204.s001.doc]

(A) Average AUCs for 18 diseases affected by *α* value when *β=*4

(B) Average AUCs for 18 diseases affected by *β* value when *α=*4

(C) Average AUCs for 18 diseases when changing *k* value

**Figure S1** **Prediction performance affected by *α* value, *β* value, and *k* value.**

According to the weight assignment strategy based on miRNA family or cluster, *α* (*β*) is a factor for adjusting the weight of the members of same family (cluster). Since *α* (*β*) appears in the denominator of the weight calculation formula, if *α* (*β*) is too small, the corresponding weight will be greater. At this time, the prediction result is excessively biased towards those miRNAs which belong to same family (cluster). If *α* (*β*) is too great, the prediction result overlooks the affect of the members of same family (cluster). Therefore, it is essential to find the suitable *α* and *β* values. The different *α* values from 1 to 10 and the different *β* values from 1 to 10 were investigated by performing the 5-fold cross validation. HDMP achieved the highest prediction performance when *α*=4 and *β=*4. SF1A shows average AUC values affected by *α* value when *β=*4. SF1B shows average AUC values affected by *β* value when *α=*4.

HDMP’s average AUC values for 18 human diseases affected by *k* value in 5-fold cross validation are illustrated in SF1C. The parameter *k* is changed from 1 to 50. Evidently, the highest prediction performance is achieved when *k* is 20.
