# Supplementary material for: Prediction of microRNAs Associated with Human Diseases Based on Weighted k Most Similar Neighbors
Source: PLoS One. 2013 Aug 8;8(8):e70204. doi: 10.1371/journal.pone.0070204 (PMC3738541; doi:10.1371/journal.pone.0070204)
Supplement: Table S1 — Prediction results of HDMP with different functional similarity measurements. (DOC) [file pone.0070204.s002.doc]

**Table S1 Prediction results of HDMP with different functional similarity measurements.**

| Disease name | AUC | |
| --- | --- | --- |
|  | HDMP’s measurement | Wang’s measurement |
| Acute myeloid leukemia | 0.822 | 0.805 |
| Adenoviridae infections | 0.686 | 0.670 |
| Breast neoplasms | 0.819 | 0.801 |
| Colorectal neoplasms | 0.785 | 0.766 |
| Glioblastoma | 0.887 | 0.868 |
| Heart failure | 0.797 | 0.767 |
| Hepatocellular carcinoma | 0.785 | 0.768 |
| Lung neoplasms | 0.899 | 0.882 |
| Lupus vulgaris | 0.681 | 0.661 |
| Medulloblastoma | 0.799 | 0.762 |
| Melanoma | 0.842 | 0.824 |
| Ovarian neoplasms | 0.836 | 0.806 |
| Pancreatic neoplasms | 0.922 | 0.904 |
| Prostatic neoplasms | 0.884 | 0.867 |
| Renal cell carcinoma | 0.828 | 0.797 |
| Squamous cell carcinoma | 0.812 | 0.789 |
| Stomach neoplasms | 0.866 | 0.845 |
| Urinary bladder neoplasms | 0.895 | 0.873 |
