# Supplementary material for: Prediction of microRNAs Associated with Human Diseases Based on Weighted k Most Similar Neighbors
Source: PLoS One. 2013 Aug 8;8(8):e70204. doi: 10.1371/journal.pone.0070204 (PMC3738541; doi:10.1371/journal.pone.0070204)
Supplement: Table S2 — Prediction results for incorporating miRNA family and cluster respectively. (DOC) [file pone.0070204.s003.doc]

**Table S2 Prediction results for incorporating miRNA family and cluster respectively**.

| Disease name | AUC | | |
| --- | --- | --- | --- |
|  | without family and cluster | with family | with cluster |
| Acute myeloid leukemia | 0.797 | 0.819 | 0.821 |
| Adenoviridae infections | 0.651 | 0.681 | 0.683 |
| Breast neoplasms | 0.788 | 0.812 | 0.817 |
| Colorectal neoplasms | 0.742 | 0.782 | 0.774 |
| Glioblastoma | 0.852 | 0.881 | 0.884 |
| Heart failure | 0.764 | 0.795 | 0.787 |
| Hepatocellular carcinoma | 0.743 | 0.781 | 0.783 |
| Lung neoplasms | 0.861 | 0.892 | 0.895 |
| Lupus vulgaris | 0.643 | 0.671 | 0.679 |
| Medulloblastoma | 0.769 | 0.796 | 0.792 |
| Melanoma | 0.805 | 0.839 | 0.837 |
| Ovarian neoplasms | 0.799 | 0.835 | 0.833 |
| Pancreatic neoplasms | 0.888 | 0.917 | 0.917 |
| Prostatic neoplasms | 0.861 | 0.882 | 0.881 |
| Renal cell carcinoma | 0.795 | 0.822 | 0.818 |
| Squamous cell carcinoma | 0.786 | 0.809 | 0.803 |
| Stomach neoplasms | 0.841 | 0.857 | 0.853 |
| Urinary bladder neoplasms | 0.859 | 0.891 | 0.893 |
