# Supplementary material for: Prediction of microRNAs Associated with Human Diseases Based on Weighted k Most Similar Neighbors
Source: PLoS One. 2013 Aug 8;8(8):e70204. doi: 10.1371/journal.pone.0070204 (PMC3738541; doi:10.1371/journal.pone.0070204)
Supplement: Table S3 — The top 50 prostatic neoplasms-related miRNA candidates in the ranked list. (1) ‘literature’ means that there is a literature to support that the miRNA is upregulated or downregulated in human prostatic neoplasm, as compared with normal prostatic tissue. (2) With analysis of the microarray data sets, a miRNA is considered to potentially have different express levels in prostatic cancer when compared to normal tissues. This kind of miRNAs is labeled by ‘dbDEMC’. (3) ‘HMDD’ means that a miRNA is a newly reported prostatic neoplasms-related miRNA which is collected by the latest version of human miRNA-disease database HMDD. (4) ‘miR2Disease’ means that a miRNA is included in the manually curated miRNA-disease association database, miR2Disease. (5) ‘higher RWRMDA’ means a miRNA has higher rank in the ranked list of RWRMDA. (6) ‘higher FCS’ means a miRNA has greater functional consistency score (FCS) among their target genes and the known target genes associated with prostatic neoplasms. (7) ‘higher Jiang’ means a miRNA has higher rank in the ranked list of Jiang's method. (DOC) [file pone.0070204.s004.doc]

**Table S3** **The top 50 prostatic neoplasms-related miRNA candidates in the ranked list.** (1) ‘literature’ means that there is a literature to support that the miRNA is upregulated or downregulated in human prostatic neoplasm, as compared with normal prostatic tissue. (2) With analysis of the microarray data sets, a miRNA is considered to potentially have different express levels in prostatic cancer when compared to normal tissues. This kind of miRNAs is labeled by ‘dbDEMC’. (3) ‘HMDD’ means that a miRNA is a newly reported prostatic neoplasms-related miRNA which is collected by the latest version of human miRNA-disease database HMDD. (4) ‘miR2Disease’ means that a miRNA is included in the manually curated miRNA-disease association database, miR2Disease. (5) ‘higher RWRMDA’ means a miRNA has higher rank in the ranked list of RWRMDA. (6) ‘higher FCS’ means a miRNA has greater functional consistency score (FCS) among their target genes and the known target genes associated with prostatic neoplasms. (7) ‘higher Jiang’ means a miRNA has higher rank in the ranked list of Jiang’s method.

| miRNA name | Description | Details |
| --- | --- | --- |
|
| hsa-mir-429 | higher RWRMDA, higher Jiang | Hsa-mir-429 is ranked No. 2 and No. 1 by RWRMDA and Jiang’s method respectively [1,2]. |
| hsa-mir-9 | dbDEMC, literature | With the significance analysis of the microarrays, hsa-mir-9 is identified as a potential miRNA downregulated in prostatic cancer when compared to normal tissues [3].  An association of has-mir-9 with the prostate cancer grade is observed [4]. |
| hsa-mir-142 | higher FCS | Hsa-mir-142 has higher functional consistency score (0.789) among their target genes and the known target genes associated with prostate cancer [5]. It is ranked No. 48 by FCS method. |
| hsa-let-7i | dbDEMC | With the significance analysis of the microarrays, hsa-let-7i is identified as a potential miRNA downregulated in prostatic cancer when compared to normal tissues [3]. |
| hsa-mir-155 | dbDEMC | With the significance analysis of the microarrays, hsa-mir-155 is identified as a potential miRNA downregulated in prostatic cancer when compared to normal tissues [3]. |
| hsa-mir-34b | dbDEMC | With the significance analysis of the microarrays, hsa-mir-34b is identified as a potential miRNA downregulated in prostatic cancer when compared to normal tissues [3]. |
| hsa-mir-19a | dbDEMC | With the significance analysis of the microarrays, hsa-mir-19a is identified as a potential miRNA downregulated in prostatic cancer when compared to normal tissues [3]. |
| hsa-mir-92a | HMDD, miR2Disease | Hsa-mir-92a is a new reported prostatic neoplasms-related miRNA after the version of human-miRNA association database HMDD released on 1 January 2012 [6].  Hsa-mir-92a is included in the manually curated miRNA-disease relationship database, miR2Disease. It means hsa-mir-92a is really associated with prostatic neoplasms [7]. |
| hsa-mir-210 | miR2Disease | Hsa-mir-210 is included in the manually curated miRNA-disease relationship database, miR2Disease. It means hsa-mir-210 is really associated with prostatic neoplasms [7]. |
| hsa-mir-19b | dbDEMC, miR2Disease | With the significance analysis of the microarrays, hsa-mir-19b is identified as a potential miRNA downregulated in prostatic cancer when compared to normal tissues [3].  Hsa-mir-19b is included in the manually curated miRNA-disease relationship database, miR2Disease. It means hsa-mir-19b is really associated with prostatic neoplasms [7]. |
| hsa-mir-224 | dbDEMC, miR2Disease | With the significance analysis of the microarrays, hsa-mir-224 is identified as a potential miRNA downregulated in prostatic cancer when compared to normal tissues [3].  Hsa-mir-224 is included in the manually curated miRNA-disease relationship database, miR2Disease. It means hsa-mir-224 is really associated with prostatic neoplasms [7]. |
| hsa-let-7f | dbDEMC, miR2Disease | With the significance analysis of the microarrays, hsa-let-7f is identified as a potential miRNA downregulated in prostatic cancer when compared to normal tissues [3].  Hsa-let-7f is included in the manually curated miRNA-disease relationship database, miR2Disease. It means hsa-let-7f is really associated with prostatic neoplasms [7]. |
| hsa-mir-199b | dbDEMC, HMDD, miR2Disease | With the significance analysis of the microarrays, hsa-mir-199b is identified as a potential miRNA downregulated in prostatic cancer when compared to normal tissues [3].  Hsa-mir-199b is a new reported prostatic neoplasms-related miRNA after the version of human-miRNA association database HMDD released on 1 January 2012 [6].  Hsa-mir-199b is included in the manually curated miRNA-disease relationship database, miR2Disease. It means hsa-mir-199b is really associated with prostatic neoplasms [7]. |
| hsa-mir-181a | dbDEMC, miR2Disease | With the significance analysis of the microarrays, hsa-mir-181a is identified as a potential miRNA downregulated in prostatic cancer when compared to normal tissues [3].  Hsa-mir-181a is included in the manually curated miRNA-disease relationship database, miR2Disease. It means hsa-mir-181a is really associated with prostatic neoplasms [7]. |
| hsa-mir-29a | dbDEMC, HMDD, miR2Disease | With the significance analysis of the microarrays, hsa-mir-29a is identified as a potential miRNA downregulated in prostatic cancer when compared to normal tissues [3].  Hsa-mir-29a is a new reported prostatic neoplasms-related miRNA after the version of human-miRNA association database HMDD released on 1 January 2012 [6].  Hsa-mir-29a is included in the manually curated miRNA-disease relationship database, miR2Disease. It means hsa-mir-29a is really associated with prostatic neoplasms [7]. |
| hsa-let-7e | dbDEMC | With the significance analysis of the microarrays, hsa-let-7e is identified as a potential miRNA downregulated in prostatic cancer when compared to normal tissues [3]. |
| hsa-mir-107 | HMDD | Hsa-mir-107 is a new reported prostatic neoplasms-related miRNA after the version of human-miRNA association database HMDD released on 1 January 2012 [6]. |
| hsa-mir-18a | higher RWRMDA, higher FCS | Hsa-mir-18a is ranked No. 15 by RWRMDA [1]. Hsa-mir-18a has higher FCS score (0.769) and it is ranked No. 92 by FCS method. |
| hsa-let-7g | dbDEMC, miR2Disease | With the significance analysis of the microarrays, hsa-let-7g is identified as a potential miRNA downregulated in prostatic cancer when compared to normal tissues [3].  Hsa-let-7g is included in the manually curated miRNA-disease relationship database, miR2Disease. It means hsa-let-7g is really associated with prostatic neoplasms [7]. |
| hsa-let-7b | dbDEMC, HMDD, miR2Disease | With the significance analysis of the microarrays, hsa-let-7b is identified as a potential miRNA upregulated in prostatic cancer when compared to normal tissues [3].  Hsa-let-7b is a new reported prostatic neoplasms-related miRNA after the version of human-miRNA association database HMDD released on 1 January 2012 [6].  Hsa-let-7b is included in the manually curated miRNA-disease relationship database, miR2Disease. It means hsa-let-7b is really associated with prostatic neoplasms [7]. |
| hsa-mir-150 | dbDEMC, literature | With the significance analysis of the microarrays, hsa-mir-150 is identified as a potential miRNA downregulated in prostatic cancer when compared to normal tissues [3].  Hsa-mir-150 has been implicated in prostate cancer development [8]. |
| hsa-mir-338 | dbDEMC | With the significance analysis of the microarrays, hsa-mir-338 is identified as a potential miRNA downregulated in prostatic cancer when compared to normal tissues [3]. |
| hsa-mir-103 | dbDEMC, miR2Disease | With the significance analysis of the microarrays, hsa-mir-103 is identified as a potential miRNA downregulated in prostatic cancer when compared to normal tissues [3].  Hsa-mir-103 is included in the manually curated miRNA-disease relationship database, miR2Disease. It means hsa-mir-103 is really associated with prostatic neoplasms [7]. |
| hsa-mir-15b | dbDEMC, HMDD | With the significance analysis of the microarrays, hsa-mir-15b is identified as a potential miRNA downregulated in prostatic cancer when compared to normal tissues [3].  Hsa-mir-15b is a new reported prostatic neoplasms-related miRNA after the version of human-miRNA association database HMDD released on 1 January 2012 [6]. |
| hsa-mir-31 | dbDEMC, HMDD, miR2Disease | With the significance analysis of the microarrays, hsa-mir-31 is identified as a potential miRNA downregulated in prostatic cancer when compared to normal tissues [3].  Hsa-mir-31 is a new reported prostatic neoplasms-related miRNA after the version of human-miRNA association database HMDD released on 1 January 2012 [6].  Hsa-mir-31 is included in the manually curated miRNA-disease relationship database, miR2Disease. It means hsa-mir-31 is really associated with prostatic neoplasms [7]. |
| hsa-mir-24 | dbDEMC, miR2Disease | With the significance analysis of the microarrays, hsa-mir-24 is identified as a potential miRNA downregulated in prostatic cancer when compared to normal tissues [3].  Hsa-mir-24 is included in the manually curated miRNA-disease relationship database, miR2Disease. It means hsa-mir-24 is really associated with prostatic neoplasms [7]. |
| hsa-mir-29c | dbDEMC | With the significance analysis of the microarrays, hsa-mir-29c is identified as a potential miRNA downregulated in prostatic cancer when compared to normal tissues [3]. |
| hsa-mir-30b | dbDEMC, miR2Disease | With the significance analysis of the microarrays, hsa-mir-30b is identified as a potential miRNA downregulated in prostatic cancer when compared to normal tissues [3].  Hsa-mir-30b is included in the manually curated miRNA-disease relationship database, miR2Disease. It means hsa-mir-30b is really associated with prostatic neoplasms [7]. |
| hsa-mir-125a | dbDEMC, miR2Disease | With the significance analysis of the microarrays, hsa-mir-125a is identified as a potential miRNA downregulated in prostatic cancer when compared to normal tissues [3].  Hsa-mir-125a is included in the manually curated miRNA-disease relationship database, miR2Disease. It means hsa-mir-125a is really associated with prostatic neoplasms [7]. |
| hsa-mir-18b | higher RWRMDA | Hsa-mir-18b is ranked No. 45 by RWRMDA [1]. |
| hsa-mir-20b | Higher FCS | Hsa-mir-20b has higher functional consistency score (0.843) among their target genes and the known target genes associated with prostate cancer [5]. It is ranked No. 5 by FCS method. |
| hsa-mir-30d | dbDEMC | With the significance analysis of the microarrays, hsa-mir-30d is identified as a potential miRNA downregulated in prostatic cancer when compared to normal tissues [3]. |
| hsa-mir-451 | literature | Hsa-mir-451 is found to be up-regulated in the metastatic prostate cancer xenografts, relative to their non-metastatic counterparts [9]. |
| hsa-mir-152 | dbDEMC | With the significance analysis of the microarrays, hsa-mir-152 is identified as a potential miRNA downregulated in prostatic cancer when compared to normal tissues [3]. |
| hsa-mir-215 | dbDEMC | With the significance analysis of the microarrays, hsa-mir-215 is identified as a potential miRNA upregulated in prostatic cancer when compared to normal tissues [3]. |
| hsa-mir-130a | dbDEMC, HMDD | With the significance analysis of the microarrays, hsa-mir-130a is identified as a potential miRNA downregulated in prostatic cancer when compared to normal tissues [3].  Hsa-mir-130a is a new reported prostatic neoplasms-related miRNA after the version of human-miRNA association database HMDD released on 1 January 2012 [6]. |
| hsa-mir-499 | higher RWRMDA | Hsa-mir-499 is ranked No. 42 by RWRMDA method [3]. |
| hsa-mir-206 | dbDEMC | With the significance analysis of the microarrays, hsa-mir-206 is identified as a potential miRNA downregulated in prostatic cancer when compared to normal tissues [3]. |
| hsa-mir-192 | dbDEMC | With the significance analysis of the microarrays, hsa-mir-192 is identified as a potential miRNA downregulated in prostatic cancer when compared to normal tissues [3]. |
| hsa-mir-335 | literature | Hsa-mir-335 is significantly down-regulated in prostate cancer cell lines than in the normal prostate cell line [10]. |
| hsa-mir-365 | literature | Hsa-mir-365 was found to be significantly differentially downregulated in LNCaP versus LNCaP-LNO cells [11]. |
| hsa-mir-30a | miR2Disease | Hsa-mir-30a is included in the manually curated miRNA-disease relationship database, miR2Disease. It means hsa-mir-30a is really associated with prostatic neoplasms [7]. |
| hsa-mir-302a | dbDEMC | With the significance analysis of the microarrays, hsa-mir-302a is identified as a potential miRNA upregulated in prostatic cancer when compared to normal tissues [3]. |
| hsa-mir-212 | literature | It is confirmed that hsa-mir-212 targets multiple signaling pathways in prostate cancer [12]. |
| hsa-mir-372 | dbDEMC | With the significance analysis of the microarrays, hsa-mir-372 is identified as a potential miRNA upregulated in prostatic cancer when compared to normal tissues [3]. |
| hsa-mir-197 | dbDEMC | With the significance analysis of the microarrays, hsa-mir-197 is identified as a potential miRNA downregulated in prostatic cancer when compared to normal tissues [3]. |
| hsa-mir-124 | literature | Hsa-mir-124 regulates cell growth of prostate cancer cells by targeting iASPP [13]. |
| hsa-mir-378 | HMDD | Hsa-mir-378 is a new reported prostatic neoplasms-related miRNA after the version of human-miRNA association database HMDD released on 1 January 2012. |
| hsa-mir-26b | dbDEMC, miR2Disease | With the significance analysis of the microarrays, hsa-mir-26b is identified as a potential miRNA downregulated in prostatic cancer when compared to normal tissues [3].  Hsa-mir-26b is included in the manually curated miRNA-disease relationship database, miR2Disease. It means hsa-mir-26b is really associated with prostatic neoplasms [7]. |
| hsa-mir-542 | higher RWRMDA | Hsa-mir-542 is ranked No. 25 by RWRMDA method [3]. |

**Reference**

1. Chen X, Liu M, Yan G. (2012) RWRMDA: predicting novel human microRNA-disease associations. Molecular BioSystems 8(10): 2792–2798.

2. Jiang Q, Hao Y, Wang G, Juan L, Zhang T, et al. (2010) Prioritization of disease microRNAs through a human phenome-microRNAome network. BMC Systems Biology 4(Suppl 1): S2.

3. Yang Z, Ren F, Liu C, He S, Sun G, et al. (2008) dbDEMC: a database of differentially expressed miRNAs in human cancers. BMC Genomics 11(Suppl 4): S5.

4. Wang L, Tang H, Thayanithy V, Subramanian S, Oberg AL, et al. (2009) Gene networks and microRNAs implicated in aggressive prostate cancer. Cancer Research 69(24): 9490–9497.

5. Li X, Wang Q, Zheng Y, Lv S, Ning S, et al. (2011) Prioritizing human cancer microRNAs based on genes’ functional consistency between microRNA and cancer. Nucleic Acids Res. 39: 1–10.

6. Lu M, Zhang Q, Deng M, Miao J, Guo Y, et al. (2008) An analysis of human microRNA and disease associations. PLoS One 3: e3420.

7. Jiang Q, Wang Y, Hao Y, Juan L, Teng M, et al. (2009) miR2Disease: a manually curated database for microRNA deregulation in human disease. Nucleic Acids Res. 37: D98–D104.

8. Waltering KK, Porkka KP, Jalava SE, Urbanucci A, Kohonen PJ, et al. (2011) Androgen regulation of micro-RNAs in prostate cancer. Prostate 71: 604–614.

9. Watahiki A, Wang Y, Morris J, Dennis K, O’Dwyer HM, et al. (2011) MicroRNAs associated with metastatic prostate cancer. PLoS ONE 6(9): e24950.

10. Xiong S, Lin T, Xu K, Dong W, Ling X, et al. (2013) MicroRNA-335 acts as a candidate tumor suppressor in prostate cancer. Pathol. Oncol. Res. Mar 3.

11. Ma S, Chan YP, Kwan PS, Lee TK, Yan M, et al. (2011) MicroRNA-616 induces androgen-independent growth of prostate cancer cells by suppressing expression of tissue factor pathway inhibitor TFPI-2. Cancer Research 71(2): 583–592.

12. Ramalinga M, Srivastava A, Dimtchev A, Soldin O, Li J, et al. (2012) MicroRNA-212 targets multiple signaling pathways in prostate cancer. Cancer Research, 72(8): 5028.

13. Chen J, Xiao H, Qi T, Chen Z, Zhang B. (2012) MicroRNA-124 regulate cell growth of prostate cancer cells targeting iASPP. Translational Andrology and Urology, s162.
