# Supplementary material for: Prediction of microRNAs Associated with Human Diseases Based on Weighted k Most Similar Neighbors
Source: PLoS One. 2013 Aug 8;8(8):e70204. doi: 10.1371/journal.pone.0070204 (PMC3738541; doi:10.1371/journal.pone.0070204)
Supplement: Table S4 — The top 50 breast neoplasms-related miRNA candidates in the ranked list. (1) ‘literature’ means that there is a literature to support that the miRNA is upregulated or downregulated in human breast neoplasm, as compared with normal breast tissue. (2) With analysis of the microarray data sets, a miRNA is considered to potentially have different express levels in breast cancer when compared to normal tissues. This kind of miRNAs is labeled by ‘dbDEMC’. (3) ‘HMDD’ means that a miRNA is a newly reported breast neoplasms-related miRNA which is collected by the latest version of human miRNA-disease database HMDD. (4) ‘miR2Disease’ means that a miRNA is included in the manually curated miRNA-disease association database, miR2Disease. (5) G2SBC is a genes-to-systems breast cancer database, which is usually used for assistant studying the breast cancer. ‘G2SBC’ means some of the top predicted target mRNAs of a miRNA are breast cancer-related genes. (6) ‘higher RWRMDA’ means a miRNA has higher rank in the ranked list of RWRMDA. (7) ‘higher FCS’ means a miRNA has greater functional consistency score (FCS) among their target genes and the known target genes associated with breast neoplasms. (DOC) [file pone.0070204.s005.doc]

**Table S4** **The top 50 breast neoplasms-related miRNA candidates in the ranked list.** (1) ‘literature’ means that there is a literature to support that the miRNA is upregulated or downregulated in human breast neoplasm, as compared with normal breast tissue. (2) With analysis of the microarray data sets, a miRNA is considered to potentially have different express levels in breast cancer when compared to normal tissues. This kind of miRNAs is labeled by ‘dbDEMC’. (3) ‘HMDD’ means that a miRNA is a newly reported breast neoplasms-related miRNA which is collected by the latest version of human miRNA-disease database HMDD. (4) ‘miR2Disease’ means that a miRNA is included in the manually curated miRNA-disease association database, miR2Disease. (5) G2SBC is a genes-to-systems breast cancer database, which is usually used for assistant studying the breast cancer. ‘G2SBC’ means some of the top predicted target mRNAs of a miRNA are breast cancer-related genes. (6) ‘higher RWRMDA’ means a miRNA has higher rank in the ranked list of RWRMDA. (7) ‘higher FCS’ means a miRNA has greater functional consistency score (FCS) among their target genes and the known target genes associated with breast neoplasms.

| miRNA name | Description | Details |
| --- | --- | --- |
|
| hsa-mir-196a | HMDD, dbDEMC, miR2disease | Hsa-mir-196a is a new reported breast neoplasms-related miRNA after the version of human-miRNA association database HMDD released on 1 January 2012 [1].  With the significance analysis of the microarrays, hsa-mir-196a is identified as a potential miRNA upregulated in breast cancer when compared to normal tissues [2].  Hsa-mir-196a is included in the manually curated miRNA-disease relationship database, miR2Disease. It means hsa-mir-196a is really associated with breast neoplasms [3]. |
| hsa-mir-16 | HMDD, dbDEMC | Hsa-mir-16 is a new reported breast neoplasms-related miRNA after the version of human-miRNA association database HMDD released on 1 January 2012 [1].  With the significance analysis of the microarrays, hsa-mir-16 is identified as a potential miRNA upregulated in breast cancer when compared to normal tissues [2]. |
| hsa-let-7i | HMDD, dbDEMC, miR2disease | Hsa-let-7i is a new reported breast neoplasms-related miRNA after the version of human-miRNA association database HMDD released on 1 January 2012 [1].  With the significance analysis of the microarrays, hsa-let-7i is identified as a potential miRNA downregulated in breast cancer when compared to normal tissues [2].  Hsa-let-7i is included in the manually curated miRNA-disease relationship database, miR2Disease. It means hsa-let-7i is really associated with breast neoplasms [3]. |
| hsa-mir-148a | HMDD, dbDEMC, miR2disease | Hsa-mir-148a is a new reported breast neoplasms-related miRNA after the version of human-miRNA association database HMDD released on 1 January 2012 [1].  With the significance analysis of the microarrays, hsa-mir-148a is identified as a potential miRNA upregulated in breast cancer when compared to normal tissues [2].  Hsa-mir-148a is included in the manually curated miRNA-disease relationship database, miR2Disease. It means hsa-mir-148a is really associated with breast neoplasms [3]. |
| hsa-let-7b | HMDD, dbDEMC | Hsa-let-7b is a new reported breast neoplasms-related miRNA after the version of human-miRNA association database HMDD released on 1 January 2012 [1].  With the significance analysis of the microarrays, hsa-let-7b is identified as a potential miRNA downregulated in breast cancer when compared to normal tissues [2]. |
| hsa-mir-106a | dbDEMC | With the significance analysis of the microarrays, hsa-mir-106a is identified as a potential miRNA downregulated in breast cancer when compared to normal tissues [2]. |
| hsa-mir-99a | dbDEMC | With the significance analysis of the microarrays, hsa-mir-99a is identified as a potential miRNA downregulated in breast cancer when compared to normal tissues [2]. |
| hsa-mir-101 | dbDEMC, miR2disease | With the significance analysis of the microarrays, hsa-mir-101 is identified as a potential miRNA downregulated in breast cancer when compared to normal tissues [2].  Hsa-mir-101 is included in the manually curated miRNA-disease relationship database, miR2Disease. It means hsa-mir-101 is really associated with breast neoplasms [3]. |
| hsa-mir-191 | HMDD, dbDEMC | Hsa-mir-191 is a new reported breast neoplasms-related miRNA after the version of human-miRNA association database HMDD released on 1 January 2012 [1].  With the significance analysis of the microarrays, hsa-mir-191 is identified as a potential miRNA downregulated in breast cancer when compared to normal tissues [2]. |
| hsa-mir-142 | higher FCS, higher RWRMDA | Hsa-mir-142 has higher functional consistency score (0.781) among their target genes and the known target genes associated with prostate cancer [4]. It is ranked No. 73 by FCS method.  Hsa-mir-142 is ranked No. 46 by RWRMDA [5]. |
| hsa-mir-29c | HMDD, dbDEMC, miR2disease | Hsa-mir-29c is a new reported breast neoplasms-related miRNA after the version of human-miRNA association database HMDD released on 1 January 2012 [1].  With the significance analysis of the microarrays, hsa-mir-29c is identified as a potential miRNA downregulated in breast cancer when compared to normal tissues [2].  Hsa-mir-29c is included in the manually curated miRNA-disease relationship database, miR2Disease. It means hsa-mir-29c is really associated with breast neoplasms [3]. |
| hsa-mir-150 | dbDEMC | With the significance analysis of the microarrays, hsa-mir-150 is identified as a potential miRNA downregulated in breast cancer when compared to normal tissues [2]. |
| hsa-mir-181c | dbDEMC | With the significance analysis of the microarrays, hsa-mir-181c is identified as a potential miRNA upregulated in breast cancer when compared to normal tissues [2]. |
| hsa-mir-27b | HMDD, dbDEMC | Hsa-mir-27b is a new reported breast neoplasms-related miRNA after the version of human-miRNA association database HMDD released on 1 January 2012 [1].  With the significance analysis of the microarrays, hsa-mir-27b is identified as a potential miRNA downregulated in breast cancer when compared to normal tissues [2]. |
| hsa-mir-99b | dbDEMC | With the significance analysis of the microarrays, hsa-mir-99b is identified as a potential miRNA downregulated in breast cancer when compared to normal tissues [2]. |
| hsa-mir-130a | dbDEMC | With the significance analysis of the microarrays, hsa-mir-130a is identified as a potential miRNA downregulated in breast cancer when compared to normal tissues [2]. |
| hsa-mir-15b | dbDEMC | With the significance analysis of the microarrays, hsa-mir-15b is identified as a potential miRNA upregulated in breast cancer when compared to normal tissues [2]. |
| hsa-mir-98 | dbDEMC, miR2disease | With the significance analysis of the microarrays, hsa-mir-98 is identified as a potential miRNA downregulated in breast cancer when compared to normal tissues [2].  Hsa-mir-98 is included in the manually curated miRNA-disease relationship database, miR2Disease. It means hsa-mir-98 is really associated with breast neoplasms [3]. |
| hsa-mir-192 | dbDEMC | With the significance analysis of the microarrays, hsa-mir-192 is identified as a potential miRNA upregulated in breast cancer when compared to normal tissues [2]. |
| hsa-mir-92b | dbDEMC | With the significance analysis of the microarrays, hsa-mir-92b is identified as a potential miRNA upregulated in breast cancer when compared to normal tissues [2]. |
| hsa-mir-30e | higher FCS, higher RWRMDA | Hsa-mir-30e has higher functional consistency score (0.795) among their target genes and the known target genes associated with prostate cancer [4]. It is ranked No. 49 by FCS method.  Hsa-mir-30e is ranked No. 7 by RWRMDA [5]. |
| hsa-mir-193a | HMDD, dbDEMC | Hsa-mir-193a is a new reported breast neoplasms-related miRNA after the version of human-miRNA association database HMDD released on 1 January 2012 [1].  With the significance analysis of the microarrays, hsa-mir-193a is identified as a potential miRNA upregulated in breast cancer when compared to normal tissues [2]. |
| hsa-mir-212 | dbDEMC | With the significance analysis of the microarrays, hsa-mir-212 is identified as a potential miRNA upregulated in breast cancer when compared to normal tissues [2]. |
| hsa-mir-196b | dbDEMC | With the significance analysis of the microarrays, hsa-mir-196b is identified as a potential miRNA downregulated in breast cancer when compared to normal tissues [2]. |
| hsa-mir-378 | dbDEMC | With the significance analysis of the microarrays, hsa-mir-378 is identified as a potential miRNA downregulated in breast cancer when compared to normal tissues [2]. |
| hsa-mir-130b | dbDEMC | With the significance analysis of the microarrays, hsa-mir-130b is identified as a potential miRNA upregulated in breast cancer when compared to normal tissues [2]. |
| hsa-mir-32 | dbDEMC | With the significance analysis of the microarrays, hsa-mir-32 is identified as a potential miRNA upregulated in breast cancer when compared to normal tissues [2]. |
| hsa-mir-372 | dbDEMC | With the significance analysis of the microarrays, hsa-mir-372 is identified as a potential miRNA downregulated in breast cancer when compared to normal tissues [2]. |
| hsa-mir-23b | HMDD, dbDEMC | Hsa-mir-23b is a new reported breast neoplasms-related miRNA after the version of human-miRNA association database HMDD released on 1 January 2012 [1].  With the significance analysis of the microarrays, hsa-mir-23b is identified as a potential miRNA downregulated in breast cancer when compared to normal tissues [2]. |
| hsa-mir-152 | HMDD, dbDEMC, miR2disease | Hsa-mir-152 is a new reported breast neoplasms-related miRNA after the version of human-miRNA association database HMDD released on 1 January 2012 [1].  With the significance analysis of the microarrays, hsa-mir-152 is identified as a potential miRNA downregulated in breast cancer when compared to normal tissues [2].  Hsa-mir-152 is included in the manually curated miRNA-disease relationship database, miR2Disease. It means hsa-mir-152 is really associated with breast neoplasms [3]. |
| hsa-mir-494 | G2SBC | 16 of top 100 hsa-mir-494’s predicted target genes are the real breast cancer-related genes. It shows that the miRNA is more likely to participate in the breast cancer-related biological process [6]. |
| hsa-mir-542 | literature | Hsa-mir-542 was significantly downregulated in the human breast cancer cells [7]. |
| hsa-mir-186 | dbDEMC | With the significance analysis of the microarrays, hsa-mir-186 is identified as a potential miRNA downregulated in breast cancer when compared to normal tissues [2]. |
| hsa-mir-138 | literature | Expression analyses revealed the significant frequent silencing of hsa-mir-138 in breast cancer [8]. |
| hsa-mir-95 | dbDEMC | With the significance analysis of the microarrays, hsa-mir-95 is identified as a potential miRNA upregulated in breast cancer when compared to normal tissues [2]. |
| hsa-mir-135a | HMDD | Hsa-mir-135a is a new reported breast neoplasms-related miRNA after the version of human-miRNA association database HMDD released on 1 January 2012 [1]. |
| hsa-mir-137 | HMDD, dbDEMC | Hsa-mir-137 is a new reported breast neoplasms-related miRNA after the version of human-miRNA association database HMDD released on 1 January 2012 [1].  With the significance analysis of the microarrays, hsa-mir-137 is identified as a potential miRNA upregulated in breast cancer when compared to normal tissues [2]. |
| hsa-mir-122 | HMDD, dbDEMC | Hsa-mir-122 is a new reported breast neoplasms-related miRNA after the version of human-miRNA association database HMDD released on 1 January 2012 [1].  With the significance analysis of the microarrays, hsa-mir-122 is identified as a potential miRNA downregulated in breast cancer when compared to normal tissues [2]. |
| hsa-mir-491 | dbDEMC | With the significance analysis of the microarrays, hsa-mir-491 is identified as a potential miRNA upregulated in breast cancer when compared to normal tissues [2]. |
| hsa-mir-625 | HMDD, dbDEMC | Hsa-mir-625 is a new reported breast neoplasms-related miRNA after the version of human-miRNA association database HMDD released on 1 January 2012 [1].  With the significance analysis of the microarrays, hsa-mir-625 is identified as a potential miRNA upregulated in breast cancer when compared to normal tissues [2]. |
| hsa-mir-181d | dbDEMC, miR2disease | With the significance analysis of the microarrays, hsa-mir-181d is identified as a potential miRNA upregulated in breast cancer when compared to normal tissues [2].  Hsa-mir-181d is included in the manually curated miRNA-disease relationship database, miR2Disease. It means hsa-mir-181d is really associated with breast neoplasms [3]. |
| hsa-mir-23a | HMDD | Hsa-mir-23a is a new reported breast neoplasms-related miRNA after the version of human-miRNA association database HMDD released on 1 January 2012 [1]. |
| hsa-mir-28 | dbDEMC | With the significance analysis of the microarrays, hsa-mir-28 is identified as a potential miRNA downregulated in breast cancer when compared to normal tissues [2]. |
| hsa-mir-184 | dbDEMC | With the significance analysis of the microarrays, hsa-mir-184 is identified as a potential miRNA upregulated in breast cancer when compared to normal tissues [2]. |
| hsa-mir-449b | literature | MiR-449b is the direct transcriptional target of E2F1 which is an important transcription factor relative to breast cancer [9]. |
| hsa-mir-381 | dbDEMC | With the significance analysis of the microarrays, hsa-mir-381 is identified as a potential miRNA downregulated in breast cancer when compared to normal tissues [2]. |
| hsa-mir-708 | G2SBC | 16 of top 100 hsa-mir-708’s predicted target genes are the real breast cancer-related genes. It shows that the miRNA is more likely to participate in the breast cancer-related biological process [6]. |
| hsa-mir-128b | HMDD | Hsa-mir-128b is a new reported breast neoplasms-related miRNA after the version of human-miRNA association database HMDD released on 1 January 2012 [1]. |
| hsa-mir-129 | HMDD, dbDEMC | Hsa-mir-129 is a new reported breast neoplasms-related miRNA after the version of human-miRNA association database HMDD released on 1 January 2012 [1].  With the significance analysis of the microarrays, hsa-mir-129 is identified as a potential miRNA upregulated in breast cancer when compared to normal tissues [2]. |
| hsa-mir-423 | HMDD | Hsa-mir-423 is a new reported breast neoplasms-related miRNA after the version of human-miRNA association database HMDD released on 1 January 2012 [1]. |

**Reference**

1. Lu M, Zhang Q, Deng M, Miao J, Guo Y, et al. (2008) An analysis of human microRNA and disease associations. PLoS One 3: e3420.

2. Yang Z, Ren F, Liu C, He S, Sun G, et al. (2008) dbDEMC: a database of differentially expressed miRNAs in human cancers. BMC Genomics 11(Suppl 4): S5.

3. Jiang Q, Wang Y, Hao Y, Juan L, Teng M, et al. (2009) miR2Disease: a manually curated database for microRNA deregulation in human disease. Nucleic Acids Res. 37: D98–D104.

4. Li X, Wang Q, Zheng Y, Lv S, Ning S, et al. (2011) Prioritizing human cancer microRNAs based on genes’ functional consistency between microRNA and cancer. Nucleic Acids Res. 39: 1–10.

5. Chen X, Liu M, Yan G. (2012) RWRMDA: predicting novel human microRNA-disease associations. Molecular BioSystems 8(10): 2792–2798.

6. Mosca E, Alfieri, R, Merelli I, Viti F, Calabria A, et al. (2010) A multilevel data integration resource for breast cancer study. BMC Systems Biology 4: 76.

7. Yamamoto Y, Yoshioka Y, Minoura K, Takahashi R, Takeshita F, et al. (2011) An integrative genomic analysis revealed the relevance of microRNA and gene expression for drug-resistance in human breast cancer cells. Molecular Cancer 10: 135.

8. Miyamoto K, Ushijima T. (2009) MicroRNAs and epigenetics in human breast cancer. Cancer Res. 69(Suppl 2): 4051.

9. Yang X, Feng M, Jiang X, Wu Z, Li Z, et al. (2009) MiR-449a and miR-449b are direct transcriptional targets of E2F1 and negatively regulate pRb–E2F1 activity through a feedback loop by targeting CDK6 and CDC25A. Genes & Dev. 23: 2388–2393.
