# Supplementary material for: Prediction of microRNAs Associated with Human Diseases Based on Weighted k Most Similar Neighbors
Source: PLoS One. 2013 Aug 8;8(8):e70204. doi: 10.1371/journal.pone.0070204 (PMC3738541; doi:10.1371/journal.pone.0070204)
Supplement: Table S5 — The top 50 lung neoplasms-related miRNA candidates in the ranked list. (1) ‘literature’ means that there is a literature to support that the miRNA is upregulated or downregulated in human lung neoplasm, as compared with normal lung tissue. (2) With analysis of the microarray data sets, a miRNA is considered to potentially have different express levels in lung cancer when compared to normal tissues. This kind of miRNAs is labeled by ‘dbDEMC’. (3) ‘HMDD’ means that a miRNA is a newly reported lung neoplasms-related miRNA which is collected by the latest version of human miRNA-disease database HMDD. (4) ‘miR2Disease’ means that a miRNA is included in the manually curated miRNA-disease association database, miR2Disease. (5) ‘higher RWRMDA’ means a miRNA has higher rank in the ranked list of RWRMDA. (6) ‘higher FCS’ means a miRNA has greater functional consistency score (FCS) among their target genes and the known target genes associated with lung neoplasms. (7) ‘higher Jiang’ means a miRNA has higher rank in the ranked list of Jiang's method. (8) ‘unconfirmed’ means there is no evidence to confirm that a miRNA is potentially associated with lung neoplasms. (DOC) [file pone.0070204.s006.doc]

**Table S5** **The top 50 lung neoplasms-related miRNA candidates in the ranked list.** (1) ‘literature’ means that there is a literature to support that the miRNA is upregulated or downregulated in human lung neoplasm, as compared with normal lung tissue. (2) With analysis of the microarray data sets, a miRNA is considered to potentially have different express levels in lung cancer when compared to normal tissues. This kind of miRNAs is labeled by ‘dbDEMC’. (3) ‘HMDD’ means that a miRNA is a newly reported lung neoplasms-related miRNA which is collected by the latest version of human miRNA-disease database HMDD. (4) ‘miR2Disease’ means that a miRNA is included in the manually curated miRNA-disease association database, miR2Disease. (5) ‘higher RWRMDA’ means a miRNA has higher rank in the ranked list of RWRMDA. (6) ‘higher FCS’ means a miRNA has greater functional consistency score (FCS) among their target genes and the known target genes associated with lung neoplasms. (7) ‘higher Jiang’ means a miRNA has higher rank in the ranked list of Jiang’s method. (8) ‘unconfirmed’ means there is no evidence to confirm that a miRNA is potentially associated with lung neoplasms.

| miRNA name | Description | Details |
| --- | --- | --- |
|
| hsa-mir-16 | dbDEMC, miR2disease | With the significance analysis of the microarrays, hsa-mir-16 is identified as a potential miRNA downregulated in lung cancer when compared to normal tissues [1].  Hsa-mir-16 is included in the manually curated miRNA-disease relationship database, miR2Disease. It means hsa-mir-16 is really associated with lung neoplasms [2]. |
| hsa-mir-15a | dbDEMC | With the significance analysis of the microarrays, hsa-mir-15a is identified as a potential miRNA downregulated in lung cancer when compared to normal tissues [1]. |
| hsa-mir-27a | dbDEMC | With the significance analysis of the microarrays, hsa-mir-27a is identified as a potential miRNA upregulated in lung cancer when compared to normal tissues [1]. |
| hsa-mir-106b | dbDEMC | With the significance analysis of the microarrays, hsa-mir-106b is identified as a potential miRNA upregulated in lung cancer when compared to normal tissues [1]. |
| hsa-mir-451 | dbDEMC, miR2disease | With the significance analysis of the microarrays, hsa-mir-451 is identified as a potential miRNA downregulated in lung cancer when compared to normal tissues [1].  Hsa-mir-451 is included in the manually curated miRNA-disease relationship database, miR2Disease. It means hsa-mir-451 is really associated with lung neoplasms [2]. |
| hsa-mir-195 | dbDEMC, miR2disease | With the significance analysis of the microarrays, hsa-mir-195 is identified as a potential miRNA downregulated in lung cancer when compared to normal tissues [1].  Hsa-mir-195 is included in the manually curated miRNA-disease relationship database, miR2Disease. It means hsa-mir-195 is really associated with lung neoplasms [2]. |
| hsa-mir-15b | dbDEMC | With the significance analysis of the microarrays, hsa-mir-15b is identified as a potential miRNA downregulated in lung cancer when compared to normal tissues [1]. |
| hsa-mir-25 | HMDD, dbDEMC | Hsa-mir-25 is a new reported lung neoplasms-related miRNA after the version of human-miRNA association database HMDD released on 1 January 2012 [3].  With the significance analysis of the microarrays, hsa-mir-25 is identified as a potential miRNA upregulated in lung cancer when compared to normal tissues [1]. |
| hsa-mir-141 | dbDEMC, miR2disease | With the significance analysis of the microarrays, hsa-mir-141 is identified as a potential miRNA upregulated in lung cancer when compared to normal tissues [1].  Hsa-mir-141 is included in the manually curated miRNA-disease relationship database, miR2Disease. It means hsa-mir-141 is really associated with lung neoplasms [2]. |
| hsa-mir-194 | literature | Hsa-mir-194 is a miRNA underexpressed in recurrence vs. no recurrence case groups of stage I of non-small cell lung carcinoma [4]. |
| hsa-mir-429 | dbDEMC, miR2disease | With the significance analysis of the microarrays, hsa-mir-429 is identified as a potential miRNA upregulated in lung cancer when compared to normal tissues [1].  Hsa-mir-429 is included in the manually curated miRNA-disease relationship database, miR2Disease. It means hsa-mir-429 is really associated with lung neoplasms [2]. |
| hsa-mir-130a | dbDEMC, miR2disease | With the significance analysis of the microarrays, hsa-mir-130a is identified as a potential miRNA downregulated in lung cancer when compared to normal tissues [1].  Hsa-mir-130a is included in the manually curated miRNA-disease relationship database, miR2Disease. It means hsa-mir-130a is really associated with lung neoplasms [2]. |
| hsa-mir-99a | dbDEMC, miR2disease | With the significance analysis of the microarrays, hsa-mir-99a is identified as a potential miRNA downregulated in lung cancer when compared to normal tissues [1].  Hsa-mir-99a is included in the manually curated miRNA-disease relationship database, miR2Disease. It means hsa-mir-99a is really associated with lung neoplasms [2]. |
| hsa-mir-20b | dbDEMC | With the significance analysis of the microarrays, hsa-mir-20b is identified as a potential miRNA upregulated in lung cancer when compared to normal tissues [1]. |
| hsa-mir-127 | HMDD, dbDEMC | Hsa-mir-127 is a new reported lung neoplasms-related miRNA after the version of human-miRNA association database HMDD released on 1 January 2012 [3].  With the significance analysis of the microarrays, hsa-mir-127 is identified as a potential miRNA downregulated in lung cancer when compared to normal tissues [1]. |
| hsa-mir-378 | literature | Hsa-mir-378 was significantly overexpressed in squamous cell carcinoma when compared with lung adenocarcinoma [5]. |
| hsa-mir-204 | miR2disease | Hsa-mir-204 is included in the manually curated miRNA-disease relationship database, miR2Disease. It means hsa-mir-204 is really associated with lung neoplasms [2]. |
| hsa-mir-135b | dbDEMC | With the significance analysis of the microarrays, hsa-mir-135b is identified as a potential miRNA downregulated in lung cancer when compared to normal tissues [1]. |
| hsa-mir-196b | dbDEMC | With the significance analysis of the microarrays, hsa-mir-196b is identified as a potential miRNA upregulated in lung cancer when compared to normal tissues [1]. |
| hsa-mir-138 | literature | Hsa-mir-138 is downregulated in lung cancer in never-smokers [4]. |
| hsa-mir-99b | literature | Hsa-mir-99b was expressed differently in the non-small cell lung carcinoma [6]. |
| hsa-mir-342 | dbDEMC | With the significance analysis of the microarrays, hsa-mir-342 is identified as a potential miRNA downregulated in lung cancer when compared to normal tissues [1]. |
| hsa-mir-296 | dbDEMC | With the significance analysis of the microarrays, hsa-mir-296 is identified as a potential miRNA downregulated in lung cancer when compared to normal tissues [1]. |
| hsa-mir-373 | higher Jiang, higher RWRMDA | Hsa-mir-373 is ranked No. 28 and No. 33 by Jiang’s method and RWRMDA method respectively [7,8]. |
| hsa-mir-22 | HMDD, miR2disease | Hsa-mir-22 is a new reported lung neoplasms-related miRNA after the version of human-miRNA association database HMDD released on 1 January 2012 [3].  Hsa-mir-22 is included in the manually curated miRNA-disease relationship database, miR2Disease. It means hsa-mir-22 is really associated with lung neoplasms [2]. |
| hsa-mir-135a | higher FCS | Hsa-mir-135a has higher functional consistency score (0.765) among their target genes and the known target genes associated with lung cancer [5]. It is ranked No. 75 by FCS method. |
| hsa-mir-152 | dbDEMC | With the significance analysis of the microarrays, hsa-mir-152 is identified as a potential miRNA downregulated in lung cancer when compared to normal tissues [2]. |
| hsa-mir-122 | literature | Hsa-mir-122 was upregulated in 5 of 8 different lung cancer patients after diagnosis [9]. |
| hsa-mir-130b | dbDEMC | With the significance analysis of the microarrays, hsa-mir-130b is identified as a potential miRNA upregulated in lung cancer when compared to normal tissues [1]. |
| hsa-mir-23b | dbDEMC | With the significance analysis of the microarrays, hsa-mir-23b is identified as a potential miRNA downregulated in lung cancer when compared to normal tissues [1]. |
| hsa-mir-137 | dbDEMC | With the significance analysis of the microarrays, hsa-mir-137 is identified as a potential miRNA downregulated in lung cancer when compared to normal tissues [1]. |
| hsa-mir-215 | dbDEMC | With the significance analysis of the microarrays, hsa-mir-215 is identified as a potential miRNA downregulated in lung cancer when compared to normal tissues [1]. |
| hsa-mir-10a | dbDEMC | With the significance analysis of the microarrays, hsa-mir-10a is identified as a potential miRNA downregulated in lung cancer when compared to normal tissues [1]. |
| hsa-mir-151 | literature | Hsa-mir-151 is upregulated in the non-small cell lung carcinoma compared to non-tumorous tissue [4]. |
| hsa-mir-328 | dbDEMC | With the significance analysis of the microarrays, hsa-mir-328 is identified as a potential miRNA upregulated in lung cancer when compared to normal tissues [1]. |
| hsa-mir-193a | HMDD | Hsa-mir-193a is a new reported lung neoplasms-related miRNA after the version of human-miRNA association database HMDD released on 1 January 2012 [3]. |
| hsa-mir-184 | literature | Hsa-mir-184 is associated with worse survival from lung cancer across studies [10]. |
| hsa-mir-491 | literature | Hsa-mir-491 is upregulated in lung adenocarcinoma compared to squamous cell carcinoma [4]. |
| hsa-mir-335 | miR2disease | Hsa-mir-335 is included in the manually curated miRNA-disease relationship database, miR2Disease. It means hsa-mir-335 is really associated with lung neoplasms [2]. |
| hsa-mir-10b | HMDD, dbDEMC | Hsa-mir-10b is a new reported lung neoplasms-related miRNA after the version of human-miRNA association database HMDD released on 1 January 2012 [3].  With the significance analysis of the microarrays, hsa-mir-10b is identified as a potential miRNA downregulated in lung cancer when compared to normal tissues [1]. |
| hsa-mir-23a | dbDEMC | With the significance analysis of the microarrays, hsa-mir-23a is identified as a potential miRNA downregulated in lung cancer when compared to normal tissues [1]. |
| hsa-mir-193b | dbDEMC | With the significance analysis of the microarrays, hsa-mir-193b is identified as a potential miRNA upregulated in lung cancer when compared to normal tissues [1]. |
| hsa-mir-7 | miR2disease | Hsa-mir-7 is included in the manually curated miRNA-disease relationship database, miR2Disease. It means hsa-mir-7 is really associated with lung neoplasms [2]. |
| hsa-mir-449a | unconfirmed | There is no evidence to confirm that hsa-mir-449a is potentially associated with lung neoplasms. |
| hsa-mir-423 | miR2disease | Hsa-mir-423 is included in the manually curated miRNA-disease relationship database, miR2Disease. It means hsa-mir-423 is really associated with lung neoplasms [2]. |
| hsa-mir-302b | dbDEMC | With the significance analysis of the microarrays, hsa-mir-302b is identified as a potential miRNA downregulated in lung cancer when compared to normal tissues [1]. |
| hsa-mir-302d | dbDEMC | With the significance analysis of the microarrays, hsa-mir-302d is identified as a potential miRNA downregulated in lung cancer when compared to normal tissues [1]. |
| hsa-mir-449b | unconfirmed | There is no evidence to confirm that hsa-mir-449b is potentially associated with lung neoplasms. |
| hsa-mir-302c | dbDEMC | With the significance analysis of the microarrays, hsa-mir-302c is identified as a potential miRNA downregulated in lung cancer when compared to normal tissues [1]. |
| hsa-mir-128a | HMDD | Hsa-mir-128a is a new reported lung neoplasms-related miRNA after the version of human-miRNA association database HMDD released on 1 January 2012 [3]. |

**Reference**

1. Yang Z, Ren F, Liu C, He S, Sun G, et al. (2008) dbDEMC: a database of differentially expressed miRNAs in human cancers. BMC Genomics 11(Suppl 4): S5.

2. Jiang Q, Wang Y, Hao Y, Juan L, Teng M, et al. (2009) miR2Disease: a manually curated database for microRNA deregulation in human disease. Nucleic Acids Res. 37: D98–D104.

3. Lu M, Zhang Q, Deng M, Miao J, Guo Y, et al. (2008) An analysis of human microRNA and disease associations. PLoS One 3: e3420.

4. Leidinger P, Keller A, Meese E (2011) MicroRNAs-important molecules in lung cancer research. Front Genet. 2: 104.

5. Lu Y, Govindan R, Wang L, Liu P, Goodgame B, et al. (2012) MicroRNA profiling and prediction of recurrence/relapse-free survival in stage I lung cancer. Carcinogenesis 0: 1–9.

6. Yanaihara N, Caplen N, Bowman E, Seike M, Kumamoto K, et al. (2006) Unique microRNA molecular profiles in lung cancer diagnosis and prognosis. Cancer Cell 9: 189–198.

7. Jiang Q, Hao Y, Wang G, Juan L, Zhang T, et al. (2010) Prioritization of disease microRNAs through a human phenome-microRNAome network. BMC Systems Biology 4(Suppl 1): S2.

8. Chen X, Liu M, Yan G. (2012) RWRMDA: predicting novel human microRNA-disease associations. Molecular BioSystems 8(10): 2792–2798.

9. Keller A, Leidinger P, Gislefoss R, Haugen A, Langseth H, et al. (2011) Stable serum miRNA profiles as potential tool for non-invasive lung cancer diagnosis. RNA Biology 8: 506–516.

10. Landi MT, Zhao Y, Rotunno M, Koshiol J, Liu H et al. (2010) MicroRNA expression differentiates histology and predicts survival of lung cancer. Clinical Cancer Research 16: 430–441.
